# Snacking on whole almonds for 6 weeks improves endothelial function and lowers LDL cholesterol but does not affect liver fat and other cardiometabolic risk factors in healthy adults: the ATTIS study, a randomized controlled trial

**DOI:** 10.1093/ajcn/nqaa100

**Published:** 2020-05-15

**Authors:** Vita Dikariyanto, Leanne Smith, Lucy Francis, May Robertson, Eslem Kusaslan, Molly O'Callaghan-Latham, Camille Palanche, Maria D'Annibale, Dimitra Christodoulou, Nicolas Basty, Brandon Whitcher, Haris Shuaib, Geoffrey Charles-Edwards, Philip J Chowienczyk, Peter R Ellis, Sarah E E Berry, Wendy L Hall

**Affiliations:** 1 Diet and Cardiometabolic Health Research Group, Department of Nutritional Sciences, School of Life Course Sciences, Faculty of Life Sciences and Medicine, King's College London, London, UK; 2 Department of Radiology, Guy's and St Thomas’ NHS Foundation Trust, London, UK; 3 Research Centre for Optimal Health, School of Life Sciences, University of Westminster, London, UK; 4 Medical Physics, Guy's and St Thomas’ NHS Foundation Trust, London, UK; 5 Institute of Psychiatry, Psychology and Neuroscience, King's College London, London, UK; 6 School of Biomedical Engineering and Imaging Sciences, Faculty of Life Sciences and Medicine, King's College London, London, UK; 7 Department of Clinical Pharmacology, School of Cardiovascular Medicine and Sciences, Faculty of Life Sciences and Medicine, King's College London, London, UK; 8 Biopolymers Group, Departments of Biochemistry and Nutritional Sciences, Faculty of Life Sciences and Medicine, King's College London, London, UK

**Keywords:** almonds, endothelial function, liver fat, dietary intervention, cardiovascular disease, cardiometabolic disease

## Abstract

**Background:**

There is convincing evidence that daily whole almond consumption lowers blood LDL cholesterol concentrations, but effects on other cardiometabolic risk factors such as endothelial function and liver fat are still to be determined.

**Objectives:**

We aimed to investigate whether isoenergetic substitution of whole almonds for control snacks with the macronutrient profile of average snack intakes, had any impact on markers of cardiometabolic health in adults aged 30–70 y at above-average risk of cardiovascular disease (CVD).

**Methods:**

The study was a 6-wk randomized controlled, parallel-arm trial. Following a 2-wk run-in period consuming control snacks (mini-muffins), participants consumed either whole roasted almonds (*n* = 51) or control snacks (*n* = 56), providing 20% of daily estimated energy requirements. Endothelial function (flow-mediated dilation), liver fat (MRI/magnetic resonance spectroscopy), and secondary outcomes as markers of cardiometabolic disease risk were assessed at baseline and end point.

**Results:**

Almonds, compared with control, increased endothelium-dependent vasodilation (mean difference 4.1%-units of measurement; 95% CI: 2.2, 5.9), but there were no differences in liver fat between groups. Plasma LDL cholesterol concentrations decreased in the almond group relative to control (mean difference −0.25 mmol/L; 95% CI: −0.45, −0.04), but there were no group differences in triglycerides, HDL cholesterol, glucose, insulin, insulin resistance, leptin, adiponectin, resistin, liver function enzymes, fetuin-A, body composition, pancreatic fat, intramyocellular lipids, fecal SCFAs, blood pressure, or 24-h heart rate variability. However, the long-phase heart rate variability parameter, very-low-frequency power, was increased during nighttime following the almond treatment compared with control (mean difference 337 ms^2^; 95% CI: 12, 661), indicating greater parasympathetic regulation.

**Conclusions:**

Whole almonds consumed as snacks markedly improve endothelial function, in addition to lowering LDL cholesterol, in adults with above-average risk of CVD.

This trial was registered at clinicaltrials.gov as NCT02907684.

## Introduction

Cardiovascular disease (CVD) continues to be the leading cause of global mortality. The development of CVD is preceded by cumulative interrelated hemodynamic and metabolic disturbances that develop over the life course and also feature in the pathophysiological progression to type 2 diabetes (T2D) (1). Dietary guidelines have been formulated partly to mitigate the progression of cardiometabolic risk phenotypes, such as raised blood pressure, dyslipidemia, and central adiposity, by encouraging a healthy eating pattern, limiting intake of added sugars, SFAs, and sodium, and choosing nutrient-dense foods in all food groups (2, 3). Most people in the United States and United Kingdom consume ≥2 snacks/d, contributing ∼20–25% of energy intake on average (4, 5). Data derived from respondents to the UK National Diet and Nutrition Survey 2008–2012 revealed that the average snack nutrient profile had 14% of energy as saturated fats and 23% of energy as sugars (predominantly added sugars) (6), exceeding dietary reference values for upper limits of their intake. Therefore, snacks present an easily modifiable target for improving overall diet quality.

Whole nuts, for example, almonds, which are mainly eaten as snacks, are encouraged as part of recommended healthy eating patterns because they are rich in protein, dietary fiber, unsaturated fatty acids, and micronutrients (vitamin E, riboflavin, niacin, and magnesium) (7, 8), and could displace other snack foods that are rich in SFAs, refined starch, and added sugar, and low in fiber. Inclusion of almonds in the diet is associated with reduced risk of CVD and T2D, and higher intakes can lower plasma LDL cholesterol and fasting blood glucose concentrations without leading to any increase in body weight (9, 10). Almond skin is a source of nonnutrient bioactives, for example, (poly)phenolic compounds, that can play a role in the mechanism of CVD prevention (11, 12). Almonds also contain significant amounts of l-arginine, the biological precursor of the potent vasodilator, nitric oxide, and they are a natural source of phytosterols, which can contribute to the LDL cholesterol–lowering properties of almonds to a limited extent (13). However, the impact of regular whole almond consumption on endothelial function, a key factor in the initiation, progression, and disease manifestation of atherosclerosis, is not yet known. Endothelial function is adversely affected by chronic low-grade inflammation and increased oxidative stress, which are pathological features associated with obesity, fatty liver, and insulin resistance. Displacement of SFAs and refined carbohydrates from typical snack products consumed in industrialized countries with unsaturated fats, protein, and fiber from whole almonds, could potentially reduce liver fat, which can subsequently impact cardiometabolic risk.

This dietary intervention trial (Almonds Trial Targeting Dietary Intervention with Snacks, or ATTIS) was designed to compare the effects of replacing habitual daily snacks with either whole almonds or control snacks with a nutritional profile matching the average UK macronutrient intakes from snacks (excluding fruit) on endothelial function and liver fat, the primary outcomes. Secondary outcomes included blood glucose, insulin, lipid profile, adipokines, markers of fatty liver, body composition, blood pressure, heart rate variability, pancreatic and skeletal muscle fat, fecal SCFAs, plasma fatty acid profiles, and metabolomic profiles. It was hypothesized that substituting whole almonds for typically consumed snacks would increase endothelium-dependent vasodilation and decrease liver fat.

## Methods

### Study population

Study participants were adult men and women (aged 30–70 y), with above-average risk of developing CVD, and self-reported regular snack consumers (≥2 snacks/d) recruited from London, United Kingdom, and the surrounding area between March 2017 and January 2019. Further details are given in **Supplemental Methods**. A CVD risk score system adapted from the Framingham risk score system was used to identify volunteers (scoring ≥2) who were above-average risk (14). Inclusion and exclusion criteria are detailed in **Supplemental Table 1**. All participants gave written informed consent before enrollment.

### Study design

The ATTIS study, conducted between March 2017 and May 2019, was approved by the UK National Research Ethics Service (REC 16/LO/1910) and registered with clinicaltrials.gov (NCT02907684). The trial was run in accordance with the Declaration of Helsinki and the principles of Good Clinical Practice.

**FIGURE 1:**
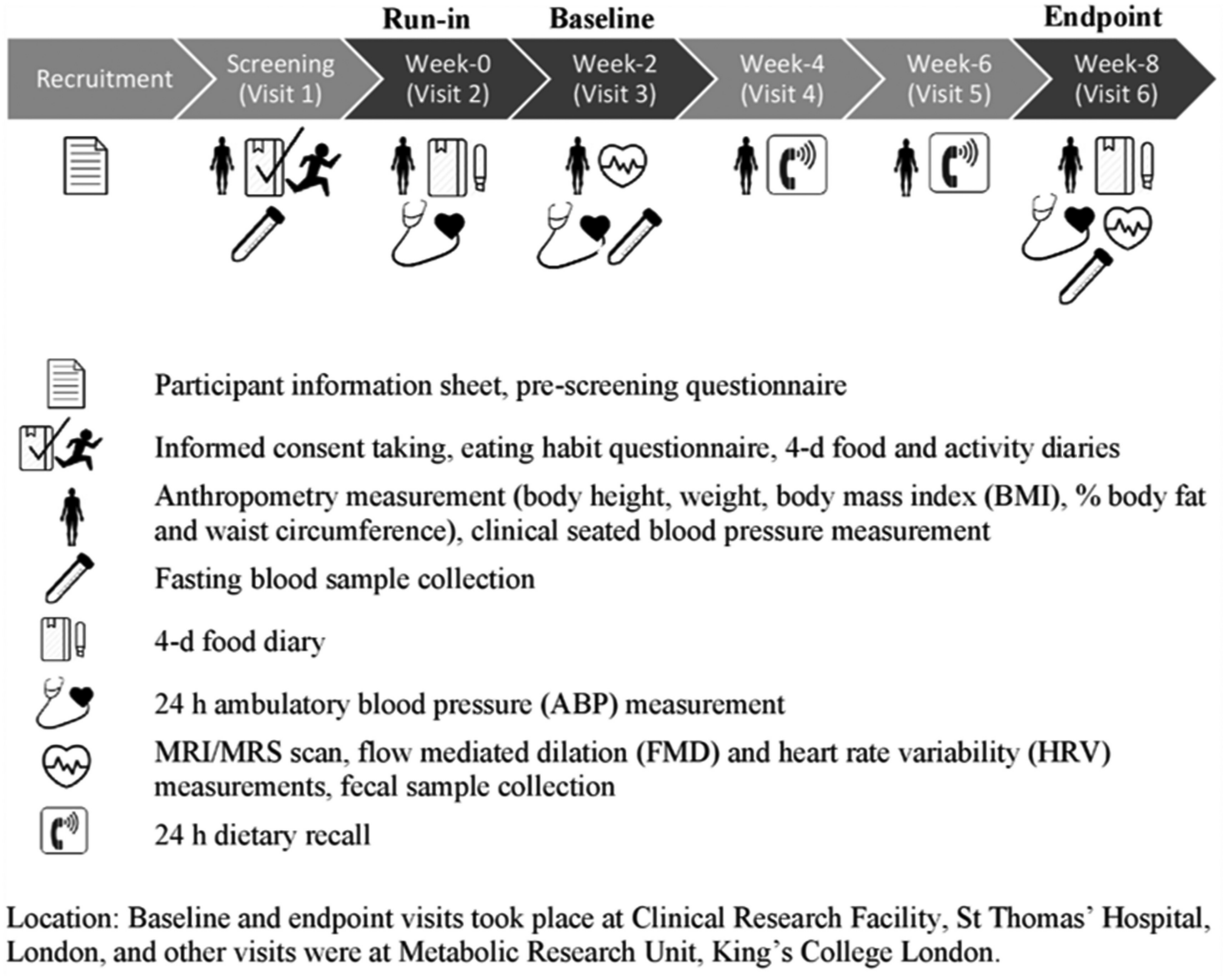
Almonds Trial Targeting Dietary Intervention with Snacks (ATTIS) study design flowchart. MRS, magnetic resonance spectroscopy.

The study was a randomized, parallel-arm design with 2 intervention groups, almonds and control, in a free-living cohort. Treatment was randomly allocated by the lead researcher using minimization software (MinimPy 0.3; Mahmoud Saghaei; http://minimpy.sourceforge.net), with age, sex, ethnicity, cardiometabolic score, and willingness to undergo MRI/magnetic resonance spectroscopy (MRS) scanning as minimization variables. A 2-wk run-in period consuming control snacks preceded random allocation to ensure that the study protocol was tolerable to individual participants prior to starting the intervention phase, and to collect baseline diet and physical activity data. Body composition, clinic blood pressure (BP), and 24-h ambulatory blood pressure (ABP) were measured; 4-d food and activity diaries were collected. Participants abstained from alcohol and strenuous activity for 24 h before baseline and end-point clinic visits, and consumed low-fat meals and no alcohol in the evening before. **Figure 1** shows the timeline of study visits and measurements. In brief, during the 6-wk intervention period, participants attended 4 visits. Baseline and end-point visits (separated by 6 wk) involved measurements of flow-mediated dilation (FMD) to assess endothelial function; MRI/MRS scan to assess liver, pancreas, and abdominal fat and myocellular lipids; ABP; heart rate variability (HRV); blood samples for glucose, insulin, lipids, and adipokines; and stool samples for fecal SCFAs. Intermediate visits (weeks 2 and 4 of the randomized intervention period) included measurements of body composition and clinic BP, and completion of a 24-h dietary recall to monitor compliance to intervention.

### Dietary intervention

The dietary intervention included a 2-wk run-in period, during which participants consumed control snacks providing 20% of estimated energy requirement (EER), followed by random allocation to either control or almond snacks at 20% of EER. Henry equations and physical activity level estimated from 4-d activity diaries were used to calculate EER (15). Sweet and savory mini-muffins were baked at the study center as the control snacks; these were formulated to provide a macronutrient profile that was representative of the average macronutrient intakes from snacks (excluding fruit) in the UK National Diet and Nutrition Survey population (6), as detailed in Supplemental Methods and **Supplemental Table 2**. Almond snacks were dry-roasted whole nonsalted almonds of the Nonpareil variety, supplied by the Almond Board of California (US grade extra no. 1). The whole almonds were weighed and packed in a daily portion for each subject. For example, 63 g (2.2 oz, or ¼ cup) of almonds were provided daily for an individual estimated to require 2000 kcal (8.37 MJ) (Supplemental Methods and **Supplemental Table 3**). During run-in and intervention periods, participants were provided with snack information sheets and dietary advice from the research dietitian and instructed to only consume study snacks between meals and to maintain their habitual mealtime eating habits and fruit consumption. All subjects were asked to avoid the consumption of additional nuts or nut products for the duration of the study.

### Outcomes

Required sample sizes were calculated for specified primary outcomes based on a 1.25% unit difference in a similar study of walnuts (SD: 1.9) in FMD (16), a measure of endothelium-dependent vasodilation (EDV), and a 2.2% unit difference in liver fat (intrahepatic lipid, IHL) between groups as being clinically important. A sample size of 50 subjects per treatment group had 90% power to detect a 1.25% unit effect of treatment on FMD with a 2-tailed α of 0.05. For liver fat, 20 subjects per group was sufficient to detect a 2.2% unit change (SD: 2.1) (17) with 90% power and a 2-tailed α of 0.05.

### Endothelial function

Following 15 min of supine rest, endothelial function was assessed as EDV of the brachial artery using the FMD technique by ultrasound (Siemens Accuson CV70), as previously reported (18). FMD was also measured post glycerol trinitrate (GTN) administration to determine endothelium-independent vasodilation. Scans were evaluated using Brachial Analyzer software (Medical Imaging Applications LLC) by a single researcher who was blinded to the identity of the participant and treatment allocation.

### Liver fat

Fifty subjects, 25 in each treatment group, agreed at screening to undergo MRI scanning. For quantification of IHL by MRI, participants were scanned from neck to foot in the supine position following an overnight fast on a 1.5-T Siemens Magnetom Aera scanner at baseline and end-point visits. The magnetic resonance protocol from which fat and water images were produced as part of the 6-point Dixon sequence was as follows (19): 8 contiguous stations covering neck-foot, acquisition time (TA) 16 s; 56 slices, field of view (FoV) 500 × 72.3%, acquired voxel size 2.2 × 2.2 × 3.5 mm, 7/8 phase and slice partial Fourier, 28.6% slice oversampling, acceleration factor 2 (CAIPIRINHA, controlled aliasing in parallel imaging results in higher acceleration; a data acquisition technique that facilitates high-resolution images for breath-hold examinations), flip angle 4°, and slice thickness 3.5 mm. To reduce motion artifacts, participants were instructed to hold their breath for 15 s while abdominal images were acquired. Liver fat was quantified using HOROS V 1.1.7 software (available at: www.horosproject.org) by a single analyst who was blinded to the participant identification code and treatment allocation of clinical data. Four regions of interest (ROIs) were placed on each slice of abdominal cavity images where the liver was visually the major sized organ in the images, avoiding blood vessels, bile ducts, and obvious artifacts (20). The ROIs were drawn in 2-point Dixon to have details of liver images visualized more clearly including vessels and ducts, and then copied and pasted into 6-point Dixon sequence. The 2-point Dixon protocol was as follows: TA 13 s; 56 slices, FoV 500 × 72.3%, acquired voxel size 1.1 × 1.1 × 3.5 mm, 7/8 phase and slice partial Fourier, 28.6% slice oversampling, acceleration factor 2 (CAIPIRINHA), flip angle 12°, and slice thickness 3.5 mm. Hepatic fat fraction was calculated in each ROI by using the mean of pixel signal intensity, and IHL was calculated as the mean of all ROIs in each slice.

IHL, alongside lipid saturation/unsaturation, was also quantified by proton magnetic resonance spectroscopy (^1^H-MRS) according to previously reported methods (21). MRS spectra were analyzed on the Java-based Magnetic Resonance User Interface (jMRUI) software with the inclusion of prior knowledge to assist in identification of the peaks of interest. Prior knowledge of a 5-resonance model was applied to fit the lipid peaks: diallylic protons [-(CH_2_)n, ∼2.9 ppm], methylenic protons (-CH_2_, ∼2.3 ppm) in the α position relative to the carboxyl group, allylic protons [-(CH_2_)n, ∼2.0 ppm], IHL/methylene protons [-(CH_2_)n, ∼1.3 ppm], and methyl protons (-CH_3_, ∼0.9 ppm). The formulas were used to determine the unsaturation index, polyunsaturation index, and saturation index of the liver fat, as previously explained by Johnson et al. (21).

### Intrapancreatic lipid, intramyocellular lipids, and body composition

Intrapancreatic lipid (IPL) quantification was measured by 2-point Dixon sequence MRI scanning. One circular 1-cm^2^ ROI was drawn on the head, body, and tail regions of the pancreas. IPL was quantified from each ROI using the formula: %IPL = [F/(F + W)] × 100, where F and W are the pixel signal intensities of the fat and water images, respectively. Mean IPL was calculated as the mean of the head, body, and tail IPL. A consultant radiologist checked and confirmed the position of ROIs. Intramyocellular lipid (IMCL) and extramyocellular lipid (EMCL) of the soleus muscle of the calf were quantified by ^1^H-MRS according to previously reported methods (22). To fit the lipid peaks for IMCL and EMCL quantification, the model used was as follows (22): water resonance 4.7 ppm, choline 3.2 ppm, creatine 3.0 ppm, EMCL-CH_2_ 1.5 ppm, IMCL-CH_2_ 1.3 ppm, EMCL-CH_3_ 1.1 ppm, and IMCL-CH_3_ 0.9 ppm. IMCL and EMCL were quantified as the ratio of the methylene IMCL or EMCL peaks (IMCL-CH_2_ or EMCL-CH_2_) to internal water.

Images for the estimation of truncal visceral adipose tissue (VAT) and subcutaneous adipose tissue (SAT) volumes were obtained from the top of the acquisition (neck) to the last slice of the abdominopelvic cavity by the same 6-point Dixon MRI scanning protocol described for liver fat, and quantification was performed using artificial intelligence–based image processing software developed at the University of Westminster, London, United Kingdom. Body weight and percentage body fat by bioelectrical impedance analysis were also measured (Tanita BC-418MA; Tanita Ltd).

### Blood pressure and heart rate variability

Ambulatory blood pressure (ABP) was measured using TM-2430 ABP monitors (A&D Inc) worn for 24 h. Readings were obtained every 30 min during daytime and every 60 min at night (22:00 to 07:00). Participants kept a record of their physical activity and sleep time in a 24-h activity diary, with self-reported sleep cutoffs being used in the analysis of daytime and nighttime BP. A&D Professional Analysis software was used to analyze mean 24-h, daytime, and nighttime SBP, DBP, and pulse. OMRON M2 Basic Intellisense monitors (OMRON Healthcare UK Ltd) were used to measure clinical blood pressure according to British Hypertension Society guidelines (23).

A small, lightweight, chest-worn, wireless 2-lead ambulatory heart rate/ECG monitor (eMotion Faros 180, Mega Electronics Ltd) was fitted to measure 24-h ambulatory HRV (24 h, daytime and nighttime). Time-domain HRV parameters included the mean of the SDs of the normal-to-normal (NN) intervals (SDNN) and root mean square of successive differences of NN intervals (RMSSD). Frequency-domain HRV parameters included high-frequency (HF) power and very-low-frequency (VLF) power. Real-time HRV was recorded during a 5-min rest, a 5-min physical stressor (inflation of blood pressure monitor cuff to 200 mmHg), and a mental stressor [the Stroop color-word test (24)]. Cardiscope Analytics software (HASIBA Medical GmbH) was used to generate HRV parameters as previously described (25).

### Blood biomarkers and fecal SCFAs

Blood and fecal sample collection procedures and analysis are explained in Supplemental Methods. Fasting insulin, glucose, nonesterified fatty acids, plasma lipids [total cholesterol (TC), HDL cholesterol, LDL cholesterol, triglyceride, and calculated TC:HDL cholesterol ratio], fatty liver indicators [fetuin-A, γ-glutamyltransferase (GGT), and alanine transaminase (ALT)], clinically relevant biomarkers of metabolic dysregulation/insulin resistance (adiponectin, resistin, and leptin), plasma fatty acids, and metabolomics were analyzed using venous blood samples taken at baseline and end point. Metabolomic profiling was performed using a high‐throughput serum NMR metabolomics platform (Nightingale Health), as described by Soininen et al. (26). All blood samples were processed by laboratory technicians blinded to participant identification and treatment allocation. Fecal samples were collected at baseline and end point in a subset of participants (*n* = 18 almond group, *n* = 17 control group) and processed within 2 h before analysis by gas chromatography, as described in Supplemental Methods.

### Compliance and dietary assessment

As outlined in Figure 1, compliance with the dietary advice was verified by telephone 24-h recalls and 4-d estimated portion size food records, which were analyzed using Nutritics software (v5.026; Nutritics Ltd) and inspection of stool samples for almond particles. Compliance scores included: *1*) complete consumption of study snacks; *2*) avoidance of consumption of other snacks; and *3*) avoidance of consumption of nuts other than almonds, as assessed by 4-d food diaries and 24-h dietary recall. The scores were as follows: score 0—did not comply; score 1—partial compliance; score 2—moderate compliance; score 3—full compliance. For participants who did not complete 4-d food diaries, the compliance was checked based solely on 24-h dietary recalls. Explanations for missing data are provided in **Supplemental Figure 1**.

### Statistical analysis

Statistical analysis was performed using IBM SPSS 25. Normality of data was assessed visually using histogram and Q-Q plot of residuals. Baseline data are shown as mean value and SD, or, if not normally distributed, as median (IQR). Treatment effects are presented as adjusted marginal mean differences of change between end-point and baseline values of the 2 groups and 95% CI. A chi-square test was conducted to investigate whether there were differences in sex and ethnicity between control and almond groups at baseline. To examine whether there were differences in other baseline characteristics and also in baseline data between the two treatment groups, independent *t* tests were used for normally distributed data, and a Mann–Whitney U test was used for nonnormally distributed data. To investigate the significance of treatment effects between the 2 groups, ANCOVA was used, with change from baseline as the dependent variable, adjusting for baseline value and baseline BMI; analysis results are presented as change values from baseline generated from estimated marginal means with CI. In addition to normality checks of residuals distribution of the changes, homogeneity of variance was examined by plotting standardized residual values compared with predicted values, and also by using Levene tests. A 2-sided *P* value  <0.05 was considered to show statistical significance.

## Results

The CONSORT flow diagram, showing the flow of participants through the study, is shown in Supplemental Figure 1. Of 294 potential participants interested in taking part in the study, 109 were eligible and 107 were randomly allocated to control or almond treatments. One hundred and five participants completed the trial: 51 in the control group and 54 in the almond group. Reasons for noncompletion included gastrointestinal intolerance of almonds (*n* = 2). The baseline characteristics of study participants randomly allocated to treatment were not different between the control and almond groups (shown in **Table 1**). Approximately 30% of participants were male and 70% female, and ∼37% identified as belonging to an ethnic group other than white.

**TABLE 1:** Participant characteristics at screening for those randomized to treatment^1^

	Control, *n* = 51	Almond, *n* = 56
Age, y	56.0 ± 10.7	56.3 ± 10.3
Sex, M/F, *n*	15/36	17/39
Ethnicity (black/South Asian, Southeast Asian, and Middle Eastern/Far East/white/other), *n*	2/6/2/34/7	9/7/3/34/3
Cardiometabolic score	4.2 ± 2.1	4.5 ± 2.0
BMI, kg/m^2^	26.7 ± 4.5	27.3 ± 4.4
Waist circumference, cm	93.3 ± 12.5	93.6 ± 12.5
Body fat, %	32.7 ± 8.5	34.4 ± 8.4
cSBP, mmHg	124.4 ± 15.1	126.2 ± 17.6
cDBP, mmHg	80.6 ± 7.7	83.8 ± 10.8
Glucose, mmol/L	5.1 ± 0.6	5.1 ± 0.5
TC, mmol/L	5.6 ± 1.2	5.6 ± 1.0
TAG, mmol/L	1.2 ± 0.5	1.2 ± 0.6
LDL, mmol/L	3.5 ± 1.0	3.4 ± 0.9
HDL, mmol/L	1.6 ± 0.5	1.6 ± 0.5
TC:HDL	3.6 ± 0.9	3.7 ± 1.1

^1^Values are means ± SD or categorical totals. Ethnicity was determined by self-reporting. Chi-square test was used to examine whether there were differences in sex and ethnicity. Independent *t* test was used to examine whether there were differences in other characteristics. There were no differences between groups for any of the parameters included in this table. cDBP, clinical diastolic blood pressure; cSBP, clinical systolic blood pressure; TAG, triglyceride; TC, total cholesterol.

### Compliance with dietary intervention

Compliance with the intervention was high as evidenced by mean (±SD) compliance scores of 2.8 ± 0.3 in the almond group and 2.7 ± 0.4 in the control group, out of a possible maximum of 3. Only 80 participants completed both sets of food diaries as per instructions with satisfactory data quality, but compliance scores based on 24-h dietary recalls did not differ significantly between those who completed both food diaries adequately and those who did not [mean compliance for food diary completers in almond group 2.9 ± 0.3 (*n* = 40), in control group 2.7 ± 0.4 (*n* = 40); for food diary noncompleters in almond group 2.9 ± 0.3 (*n* = 14) and control group 2.5 ± 0.6 (*n* = 11)].

**Table 2** shows the nutrient intake of the study population as measured from 4-d diet diaries at baseline (week 0) and end of intervention from the 40 participants of each group who completed diaries at both time points. At baseline, SFAs provided 12.3% and 12.5% of energy intake, and free sugars provided 5.9% and 5.5% of energy intake for the control and almond groups, respectively. There was no difference in the change from baseline in energy intake between control and almond groups during the intervention. Relative to the control group, the almond group reduced intakes of total carbohydrates, starch, and free sugars as a percentage of energy intake by 9.3%, 7.0%, and 3.0%, respectively, and increased dietary fiber intake by 7.4%. Total fat intake as a percentage of energy intake and the ratio of unsaturated to saturated fatty acids were significantly increased in the almond group, by 10.8% and 1.3%, respectively, compared with the control group. The almond group also increased intakes of potassium, magnesium, vitamin E, and riboflavin, and lowered intake of sodium relative to the control group.

**TABLE 2:** Nutrient intakes estimated from 4-d food diaries at baseline (prior to run-in) and the final week of the dietary intervention^1^

	Control,^2^*n*_max_ = 40		Almond,^2^*n*_max_ = 40		Mean comparison between groups	*P* value
	Baseline	Change	Baseline	Change		
Energy intake,^3^ kcal/d	2088.9 ± 538.5	−5.8 (−124.7, 113.2)	1769.4 ± 475.0	−85.3 (−204.3, 33.7)	−79.5 (−251.8, 92.8)	0.361
Protein, %E	15.4 ± 3.8	0.5 (−0.5, 1.5)	15.9 ± 3.6	1.0 (0.0, 2.0)	0.5 (−0.9, 1.9)	0.466
Total carbohydrate, %E	43.3 ± 7.1	1.7 (−0.1, 3.5)	41.8 ± 6.6	−7.6 (−9.4, −5.8)	−9.3 (−11.9, −6.8)	<0.001
Starch, %E	23.9 ± 5.1	2.5 (0.9, 4.1)	23.5 ± 5.3	−4.5 (−6.1, −2.9)	−7.0 (−9.3, −4.8)	<0.001
Free sugars, %E	5.9 ± 3.8	0.4 (−0.5, 1.2)	5.5 ± 2.8	−2.6 (−3.5, −1.8)	−3.0 (−4.2, −1.8)	<0.001
Dietary fiber,^3^ g/d	23.8 ± 6.2	−1.9 (−4.5, 0.6)	20.7 ± 7.7	5.5 (3.0, 8.1)	7.4 (3.8, 11.1)	<0.001
Fat, %E	36.5 ± 6.5	−2.6 (−4.3, −0.8)	37.1 ± 6.2	8.3 (6.5, 10.0)	10.8 (8.4, 13.3)	<0.001
SFA, %E	12.3 ± 3.6	−0.6 (−1.3, 0.1)	12.5 ± 3.7	−1.4 (−2.1, −0.6)	−0.7 (−1.8, 0.3)	0.153
MUFA, %E	11.5 ± 3.4	−1.1 (−2.4, 0.0)	12.4 ± 3.7	8.6 (7.4, 9.8)	9.8 (8.1, 11.5)	<0.001
PUFA, %E	5.9 ± 2.5	−0.8 (−1.4, −0.1)	5.9 ± 1.7	2.0 (1.4, 2.6)	2.8 (1.9, 3.7)	<0.001
Unsaturated:saturated fatty acid ratio	−0.1 ± 0.6	−0.1 (−0.3, 0.1)	1.1 ± 1.1	1.1 (0.9, 1.3)	1.3 (1.0, 1.5)	<0.001
Sodium, mg	2151.2 ± 766.3	179.7 (−15.8, 375.3)	1926.1 ± 866.1	−490.8 (−686.4, −295.3)	−670.6 (−948.6, −392.6)	<0.001
Potassium,^3^ mg	3028.9 ± 936.2	−352.5 (−590.4, −114.5)	2534.7 ± 854.5	221.3 (−16.7, 459.3)	573.8 (231.0, 916.6)	0.001
Calcium,^3^ mg	868.4 ± 455.8	24.2 (−57.6, 106.0)	703.8 ± 242.5	57.3 (−24.5, 139.0)	33.1 (−84.0, 150.2)	0.575
Magnesium,^3^ mg	368.7 ± 180.9	−36.0 (−68.9, −3.0)	278.5 ± 92.1	112.6 (79.7, 145.5)	148.6 (100.9, 196.3)	<0.001
Vitamin E, mg	10.7 ± 3.7	−1.9 (−3.5, −0.4)	8.9 ± 4.0	13.5 (11.9, 15.0)	15.4 (13.2, 17.6)	<0.001
Riboflavin, mg	1.8 ± 1.6	−0.1 (−0.3, 0.0)	1.5 ± 0.8	0.4 (0.2, 0.6)	0.5 (0.3, 0.8)	<0.001
Niacin, mg	16.1 ± 8.9	−1.6 (−3.3, 0.1)	14.7 ± 9.4	−0.4 (−2.1, 1.3)	1.2 (−1.3, 3.6)	0.339

^1^Baseline data are mean ± SD. Values of change and main comparisons of changes between groups are presented as mean (95% CI) generated from estimated marginal means from ANCOVA, adjusted for baseline value and baseline BMI. ANCOVA assumptions were met. *P* < 0.05 indicates a significant difference. .

2 Data were analyzed using 40 diaries collected from each group. Missing data are due to poor quality of diet diaries or failure to complete by participant.

3 Baseline value was different between control and almond group. %E, % of energy intake.

To determine whether pre–post intervention changes were significant in the almond group but not the control group, repeated measures ANCOVA (with time as a repeated measure and BMI as covariate) showed that the decreases in carbohydrate, starch, free sugars, and sodium intakes in the almond group were significant (time × intervention group interactions are presented in **Supplemental Table 4**), whereas there were no significant changes in the control group for these nutrients. Furthermore, the increases in intakes of dietary fiber, fat, MUFAs, PUFAs, the ratio of unsaturated to saturated fatty acids, and vitamin E in the almond group were significant, but there were no significant changes in the control group. Potassium, magnesium, and riboflavin significantly increased in the almond group but also significantly decreased in the control group, although the magnitude of the changes was smaller in the control group for magnesium and riboflavin. There were no significant changes in total energy, SFA, protein, and niacin intake within either group.

### Vascular health

Vascular-related measures are shown in **Table 3**. There was a significant treatment difference in the change from baseline values for FMD. Almond consumption significantly increased FMD by 4.1% units relative to the change following control snacks (95% CI: 2.2, 5.9% units; *P <* 0.00005). There were no differential changes in baseline brachial artery diameter or in endothelium-independent vasodilation (following GTN) between groups. VLF power, a frequency domain parameter of HRV, was increased during sleep-time by 337 ms^2^ (95% CI: 12, 661 ms^2^; *P <* 0.05) following almond consumption. There were no significant group differences observed in changes in blood pressure or in 24-h HRV.

**TABLE 3:** Changes in indices of vascular function, blood pressure, and heart rate variability following randomization to almond and control snacks^1^

	Control,^2^*n*_max_ = 51		Almonds,^2^*n*_max_ = 54		Main comparison between groups^4^
	Baseline^3^	Change	Baseline^3^	Change	
Endothelium-dependent vasodilation					
FMD,^5^ %	7.0 ± 4.8	−0.8 (−2.1, 0.5)	3.6 ± 3.9	3.3 (2.0, 4.5)	4.1 (2.2, 5.9)^6^
Prehyperemia brachial artery diameter, mm	3.49 ± 0.58	0.00 (−0.09, 0.09)	3.56 ± 0.48	−0.01 (−0.09, 0.08)	−0.01 (−0.14, 0.11)
GTN, %	14.8 ± 6.5	−0.2 (−1.6, 1.3)	12.6 ± 4.9	0.7 (−0.6, 2.0)	0.9 (−1.1, 2.9)
Clinic blood pressure					
cSBP, mmHg	127.8 ± 12.9	−5.2 (−7.9, −2.6)	127.3 ± 19.3	−6.3 (−8.9, −3.7)	−1.0 (−4.7, 2.6)
cDBP, mmHg	84.6 ± 7.9	−3.2 (−5.1, −1.3)	85.5 ± 10.6	−3.5 (−5.3, −1.6)	0.2 (−2.9, 2.4)
Ambulatory blood pressure					
24-h SBP,^5^ mmHg	122.7 ± 9.7	0.4 (−2.0, 2.8)	128.0 ± 15.0	−1.0 (−3.3, 1.3)	−1.4 (−4.8, 1.9)
24-h DBP,^5^ mmHg	74.1 ± 6.2	−0.2 (−1.9, 1.5)	77.6 ± 9.3	−0.6 (−2.3, 1.0)	−0.4 (−2.8, 2.0)
24-h pulse, beats/min	71.9 ± 8.4	−0.4 (−2.2, 1.4)	72.6 ± 9.3	−1.0 (−2.8, 0.7)	−0.7 (−3.2, 1.9)
Daytime SBP,^5^ mmHg	126.1 ± 9.8	−0.1 (−2.7, 2.6)	132.6 ± 16.0	−0.4 (−2.9, 2.2)	−0.3 (−4.1, 3.5)
Daytime DBP,^5^ mmHg	76.2 ± 6.5	0.1 (−1.8, 2.1)	80.9 ± 9.9	−0.2 (−2.0, 1.7)	−0.3 (−3.1, 2.4)
Daytime pulse, beats/min	74.5 ± 8.5	0.1 (−1.9, 2.1)	75.3 ± 9.5	−1.3 (−3.3, 0.6)	−1.1 (−4.2, 1.3)
Nighttime SBP, mmHg	109.3 ± 11.9	1.2 (−1.9, 4.9)	111.6 ± 15.3	−1.0 (−4.3, 2.4)	−3.0 (−7.2, 1.3)
Nighttime DBP, mmHg	64.6 ± 7.6	−0.1 (−2.3, 2.0)	65.6 ± 8.5	−1.4 (−3.4, 0.6)	−1.3 (−4.2, 1.6)
Nighttime pulse, beats/min	61.5 ± 8.1	−0.8 (−2.7, 1.0)	63.0 ± 9.7	−0.2 (−1.9, 1.5)	0.7(−1.8, 3.2)
Heart rate variability (24 h and nighttime)					
24-h SDNN, ms	148.8 ± 36.5	−8.2 (−17.3, −0.9)	142.2 ± 35.0	−5.2 (−14.9, 4.5)	3.0 (−10.3, 16.4)
24-h rMSSD, ms	28.5 ± 8.9	1.9 (−0.6, 4.3)	30.5 ± 9.8	0.6 (−2.1, 3.2)	−1.3 (−4.9, 2.3)
Nighttime SDANN, ms	64.1 ± 22.3	−0.8 (−8.3, 6.8)	66.5 ± 33.0	1.0 (−6.4, 8.4)	1.8 (−8.8, 12.3)
Nighttime rMSSD, ms	31.3 (20.9)	1.9 (−1.3, 5.0)	33.9 (17.4)	−1.1 (−4.1, 1.9)	−3.0 (−7.3, 1.4)
Nighttime VLF, ms^2^	1664 (2720)	−293 (−523, −62)	1595 (1387)	44 (−182, 270)	337 (12, 661)^7^
Nighttime HF, ms^2^	311.5 (545.3)	0.7 (−84.1, 85.6)	356 (331)	−0.2 (−83.3, 82.8)	−10.0 (−120.0, 118.1)

^1^Values of change and main comparisons of changes between groups are presented as mean (95% CI) generated from estimated marginal means from ANCOVA.

2 Not all data were analyzed due to poor-quality read-outs. FMD and prehyperemia brachial artery diameter: *n* = 42 (control) and 47 (almond). GTN: *n* = 32 (control) and 41 (almond). 24-h SBP, 24-h DBP, 24-h pulse, daytime SBP, DBP, and pulse: *n* = 45 (control) and 49 (almond). Nighttime SBP, DBP, and pulse: *n* = 40 (control) and 46 (almond). 24-h SDNN and 24-h rMSSD: *n* = 33 (control) and 29 (almond). Nighttime SDANN: *n* = 45 (control) and 47 (almond). Nighttime rMSSD: *n* = 45 (control) and 50 (almond). Nighttime VLF and sleep-time HF: *n* = 45 (control) and 47 (almond). Nighttime SDANN: *n* = 45 (control) and 47 (almond).

3 Median (IQR) for nighttime rMSSD, VLF, and HF data because they are nonnormally distributed. Mean ± SD for other data that are normally distributed.

4 ANCOVA, adjusted for baseline outcome value and baseline BMI (mean difference in the change from baseline, almonds minus control). ANCOVA assumptions were met.

5 Baseline value was different between control and almond group; independent *t* test was used, *P* < 0.05 indicated a significant difference.

6 *P <* 0.00005.

7 *P* < 0.05. cDBP, clinic diastolic blood pressure; cSBP, clinic systolic blood pressure; DBP, diastolic blood pressure; FMD, flow-mediated dilation; GTN, glycerol trinitrate; HF, absolute power of the high-frequency band (0.15–0.04 Hz); rMSSD, root mean square of successive R-R interval differences; SBP, systolic blood pressure; SDANN, standard deviation of the average NN intervals for each 5-min segment of heart rate variability recording; SDNN, standard deviation of normal-to-normal (NN) intervals; VLF, absolute power of the very-low-frequency band (0.0033–0.04 Hz).

### Other cardiometabolic outcomes

As shown in **Table 4**, there were no treatment effects of diet on BMI, waist circumference, percentage body fat (measured by bioelectrical impedance), or truncal VAT or SAT volumes (measured by MRI). Liver fat was unaffected by treatment, as was pancreatic and skeletal muscle fat (measured by MRI and MRS). Fasting plasma non-HDL and LDL cholesterol concentrations were significantly reduced by almond snacks relative to control by −0.22 mmol/L (95% CI: −0.42, −0.01 mmol/L; *P* = 0.037) and −0.25 mmol/L (95% CI: −0.45, −0.04 mmol/L; *P* = 0.017), respectively (**Table 5**), but there were no significant treatment effects on HDL cholesterol, triacylglycerol, glucose, insulin, HOMA-IR, adipokines, and markers of fatty liver (adiponectin, leptin, resistin, fetuin-A, ALT, and GGT). **Table 6** presents fasting plasma fatty acid profiles in both groups. Plasma concentrations of oleic acid, which made up 66% of total fatty acid content of the almonds used in the study, were increased after almond consumption relative to control by 228 µmol/L (95% CI: 7, 449 µmol/L; *P* = 0.043), but no significant differences in plasma concentrations of the other main fatty acid, linoleic acid (22% of total fatty acid content of these almonds), nor any other plasma fatty acids were observed. Furthermore, there were no effects of treatment on fecal SCFA composition or total SCFA concentrations (**Table 7**). There were no clear treatment effects on metabolomic profiles (measured by NMR), although serum total ω-3 concentrations decreased by −0.04 mmol/L (*P* = 0.031) and citrate concentrations increased by 0.01 mmol/L (*P* = 0.018) in the almond group relative to the control group (see **Supplemental Table 5**).

**TABLE 4:** Body composition and measures of ectopic fat, following randomization to almond and control snacks^1^

	Control,^2^*n*_max_ = 51		Almonds,^2^*n*_max_ = 54		Main comparison between groups^4^
	Baseline^3^	Change	Baseline^3^	Change	
Physical activity by accelerometry,^5^ cpm	74.2 ± 17.1	1.5 (−4.0, 7.1)	74.6 ± 21.8	−2.4 (−7.7, 2.8)	−4.0 (−11.6, 3.6)
BMI, kg/m^2^	27.1 ± 4.4	−0.2 (−0.4, 0.0)	27.2 ± 4.5	0.1 (−0.1, 0.3)	0.2 (−0.1, 0.5)
WC, cm	93.3 ± 11.7	0.1 (−0.9, 1.2)	94.1 ± 12.2	−0.6 (−1.6, 0.5)	−0.7 (−2.2, 0.8)
Body fat, %	31.1 ± 7.7	−0.5 (−1.1, 0.0)	32.2 ± 7.7	0.3 (−0.3, 0.8)	0.8 (−0.0, 1.6)
MRI and ^1^H-MRS					
Liver fat %	2.7 (2.5)	0.4 (−0.5, 1.3)	1.7 (1.7)	1.1 (0.1, 2.0)	0.7 (−0.6, 2.0)
IHL %	2.9 (4.0)	0.1 (−1.3, 1.6)	1.7 (2.3)	−0.6 (−2.1, 0.8)	−0.8 (−2.8, 1.3)
UI	0.21 ± 0.12	0.03 (−0.06, 0.12)	0.32 ± 0.20	0.01 (−0.08, 0.10)	−0.02 (−0.15, 0.10)
PUI	0.03 (0.11)	0.02 (−0.05, 0.10)	0.04 (0.21)	0.03 (−0.04, 0.10)	0.01 (−0.09, 0.11)
SI	0.77 (0.13)	−0.03 (−0.12, 0.05)	0.70 (0.35)	−0.01 (−0.10, 0.08)	0.02 (−0.10, 0.15)
Pancreatic fat, %	10.7 (3.8)	0.1 (−1.1, 1.3)	10.7 (3.2)	0.1 (−1.1, 1.4)	0.0 (−1.7, 1.8)
SAT, mL	13,703 ± 5290	678 (−41, 1397)	12,981 ± 5264	−20 (−771, 732)	−697 (−1741, 347)
VAT, mL	3492 ± 1930	−13 (−95, 69)	3009 ± 1889	34 (−50, 118)	47 (−71, 165)
IMCL	0.037 ± 0.027	0.002 (−0.005, 0.010)	0.030 ± 0.014	−0.005 (−0.012, 0.003)	−0.007 (−0.018, 0.003)
EMCL	0.035 (0.027)	0.001 (−0.009, 0.010)	0.020 (0.015)	−0.001 (−0.011, 0.009)	−0.001 (−0.015, 0.013)

^1^Values of change and main comparison of the changes between groups are presented as mean (95% CI) generated from estimated marginal means from ANCOVA.

2 MRI/MRS scanning was planned on a subset of study participants: *n* = 50 (25 per group). Not all data were analyzed due to technical problems. Physical activity by accelerometery: *n* = 45 (control) and 50 (almond). BMI: *n* = 45 (control) and 50 (almond). WC: *n* = 49 (control) and 51 (almond). Body fat: *n* = 49 (control) and 52 (almond). Liver fat and pancreatic fat: *n* = 26 (control) and 24 (almond). IHL, UI, PUI, and SI: *n* = 22 (control) and 23 (almond). SAT: *n* = 24 (control) and 22 (almond). VAT: *n* = 23 (control) and 22 (almond). IMCL and EMCL: *n* = 23 (control) and 22 (almond).

3 Median (IQR) for liver fat, IHL, PUI, SI, pancreatic fat, and EMCL data because they are nonnormally distributed. Mean ± SD for other data that are normally distributed. Baseline biomarker values were not different between the 2 groups.

4 ANCOVA, adjusted for baseline outcome value and baseline BMI (mean difference in change from baseline, almonds minus control); there were no significant differences between groups. ANCOVA assumptions were met.

^5^Physical activity by accelerometery data were generated from heart rate variability monitoring. Cpm, counts per minute; EMCL, extramyocellular lipid; IHL, intrahepatic lipid; IMCL, intramyocellular lipid; PUI, polyunsaturation index; SAT, subcutaneous fat; SI, saturation index; UI, unsaturation index; VAT, visceral fat; WC, waist circumference; ^1^H-MRS, proton magnetic resonance spectroscopy.

**TABLE 5:** Circulating biomarkers of cardiometabolic risk following randomization to almond and control snacks^1^

	Control,^2^*n*_max_ = 51		Almonds,^2^*n*_max_ = 54		Main comparison between groups^4^
	Baseline^3^	Change	Baseline^3^	Change	
HOMA-IR	1.78 ± 1.38	0.21 (−0.17, 0.60)	1.64 ± 0.96	0.05 (−0.32, 0.41)	−0.16 (−0.70, 0.37)
Glucose, mmol/L	5.3 ± 0.6	0.02 (−0.14, 0.17)	5.3 ± 0.7	0.03 (−0.12, 0.18)	0.01 (−0.20, 0.23)
Insulin, mIU/L	7.1 ± 4.3	0.70 (−0.68, 2.07)	6.9 ± 3.5	0.08 (−1.24, 1.39)	−0.62 (−2.52, 1.28)
NEFA, mmol/L	0.66 ± 0.24	−0.03 (−0.09, 0.04)	0.64 ± 0.24	−0.00 (−0.06, 0.07)	−0.03 (−0.07, 0.12)
TC, mmol/L	5.26 ± 1.13	0.03 (−0.15, 0.20)	5.40 ± 0.93	−0.18 (−0.35, −0.02)	−0.21 (−0.45, 0.03)
TAG, mmol/L	1.17 (0.69)	−0.11 (−0.20, 0.01)	1.07 (0.73)	−0.08 (−0.17, 0.02)	0.03 (−0.11, 0.16)
Non-HDL-C, mmol/L	3.92 ± 1.16	0.11 (−0.04, 0.26)	4.00 ± 0.98	−0.11 (−0.25, 0.03	−0.22 (−0.42, −0.01)^5^
LDL-C, mmol/L	3.63 ± 1.16	0.15 (0.01, 0.30)	3.74 ± 0.91	−0.09 (−0.23, 0.05)	−0.25 (−0.45, −0.04)^5^
HDL-C, mmol/L	1.61 ± 0.45	0.04 (−0.04, 0.11)	1.66 ± 0.51	−0.04 (−0.11, −0.03)	−0.08 (−0.18, 0.03)
TC:HDL-C	3.45 ± 0.91	−0.04 (−0.15, 0.07)	3.47 ± 1.01	−0.03 (−0.14, 0.07)	0.00 (−0.15, 0.16)
Leptin, μg/L	12.50 (13.41)	−0.34 (−2.08, 1.41)	17.49 (20.24)	0.25 (−1.44, 1.94)	0.59 (−1.86, 3.03)
Adiponectin, mg/L	7.64 (5.96)	−0.16 (−0.76, 0.44)	7.92 (5.86)	−0.14 (−0.72, 0.44)	0.02 (−0.82, 0.85)
ALT, IU/L	22.00 (9.8)	−0.46 (−3.52, 2.59)	22.10 (10.10)	1.31 (−1.63, 4.25)	1.77 (−2.47, 6.01)
GGT, IU/L	12.60 (11.80)	−0.77 (−3.58, 2.05)	15.20 (11.00)	0.92 (−1.79, 3.63)	1.69 (−2.22, 5.59)
Fetuin A, mg/L	698.60 ± 132.86	−2.70 (−37.71, 32.32)	665.56 ± 136.83	15.91 (−18.08, 49.89)	18.60 (−30.33, 67.53)
Resistin, μg/L	5.25 ± 2.29	0.12 (−0.20, 0.44)	5.04 ± 1.79	−0.01 (−0.32, 0.30)	−0.13 (−0.57, 0.31)

^1^Values of change and main comparisons of the changes between groups are presented as mean (95% CI) generated from estimated marginal means from ANCOVA.

2 Not all data were analyzed due to technical problems and sample loss. HOMA-IR and glucose: *n* = 48 (control) and 53 (almond). Insulin, NEFA, TC, TAG, non-HDL-C, LDL-C, HDL-C, TC:HDL-C, ALT, GGT: *n* = 49 (control) and 53 (almond). Leptin, adiponectin, fetuin-A, and resistin: *n* = 49 (control) and 52 (almond).

3 Median (IQR) for TAG, leptin, and GGT data because they are nonnormally distributed. Mean ± SD for other data that are normally distributed. Baseline biomarker values were not different between the 2 groups.

4 ANCOVA, adjusted for baseline outcome value and baseline BMI (mean difference in change from baseline, almonds minus control); *P* < 0.05 indicated a significant difference. ANCOVA assumptions were met.

5 *P* < 0.05 indicated a significant difference for values of mean difference between 2 groups. ALT, alanine aminotransferase; GGT, γ-glutamyltransferase; HDL-C, high-density lipoprotein cholesterol; LDL-C, low-density lipoprotein cholesterol; NEFA, nonesterified fatty acid; TAG, triglyceride; TC, total cholesterol.

**TABLE 6:** Plasma fatty acid profile following randomization to almond and control snacks^1^

	Control, *n* = 48		Almonds, *n* = 53		Main comparison between groups^3^
FA, µmol/L	Baseline^2^	Change	Baseline^2^	Change	
Palmitic (16:0)	2334.3 ± 992.4	−105.6 (−322.3, 111.1)	2296.8 ± 933.1	53.6 (−152.6, 259.9)	159.2 (−140.1, 458.6)
Palmitoleic (16:1)	300.8 ± 175.8	−18.2 (−42.1, 5.7)	282.9 ± 156.7	−25.1 (−47.9, −2.4)	−7.0 (−40.0, 26.0)
Stearic (18:0)	690.4 ± 268.1	−24.9 (−76.4, 26.6)	674.6 ± 241.2	5.2 (−43.8, 54.2)	30.1 (−41.0, 101.2)
Oleic (18:1n–9)	2300.4 ± 1032.1	−111.3 (−271.1, 48.5)	2232.9 ± 915.3	116.6 (−35.5, 268.7)	227.9 (7.2, 448.7)^4^
Linoleic (18:2n–6)	2873.4 ± 1004.6	−23.2 (−247.9, 201.6)	2853.6 ± 810.4	107.7 (−124.1, 303.6)	112.9 (−197.5, 423.3)
α-Linolenic (18:3n–3)	155.9 ± 81.6	4.3 (−16.5, 25.1)	134.6 ± 67.5	−20.3 (−40.0, −0.6)	−24.6 (−53.4, 4.2)
γ-Linolenic (18:3n–6)	76.1 (76.5)	−9.5 (−21.2, 2.2)	70.8 (83.3)	−9.5 (−20.6, 1.7)	0.0 (−16.1, 16.2)
Homo-γ-linoleic (20:3n–6)	141.8 ± 65.3	−2.8 (−17.1, 11.4)	136.5 ± 60.0	0.9 (−12.7, 14.4)	3.7 (−16.0, 23.4)
Arachidonic (20:4n–6)	601.5 ± 302.3	−26.8 (−72.9, 19.4)	623.3 ± 243.4	15.0 (−28.9, 59.0)	41.8 (−22.0, 105.6)
Eicosapentaenoic (20:5n–3)	101.2 (77.3)	8.6 (−9.7, 26.9)	96.1 (81.1)	−11.6 (−29.0, 5.8)	−20.2 (−45.4, 5.1)
Docosatetraenoic (22:4n–6)	31.9 (25.3)	−1.1 (−3.5, 1.3)	29.0 (28.2)	−2.4 (−4.6, −0.1)	−1.3 (−4.6, 2.0)
Docosapentaenoic (22:5n–3)	47.9 ± 18.9	−0.4 (−4.3, 3.6)	47.0 ± 19.9	−3.6 (−7.4, 0.2)	−3.2 (−8.7, 2.2)
Docosapentaenoic (22:5n–6)	47.2 (59.8)	−0.2 (−4.9, 4.6)	49.0 (49.4)	1.3 (−3.3, 5.8)	1.5 (−5.1, 8.0)
Docosahexaenoic (22:6n–3)	184.9 ± 77.3	5.0 (−10.9, 20.9)	176.8 ± 81.0	−5.9 (−21.0, 9.2)	−10.9 (−32.8, 11.1)
Total plasma FA	9913.6 ± 3685.5	−303.4 (−1014.5, 407.7)	9741.3 ± 3275.5	201.4 (−475.2, 878.1)	504.8 (−477.4, 1487.0)

^1^Values of change and main comparisons of changes between groups are presented as mean (95% CI) generated from estimated marginal means from ANCOVA.

2 Median (IQR) for γ-linolenic (18:3n–6), eicosapentaenoic (20:5n–3), docosatetraenoic (22:4n–6), and docosapentaenoic (22:5n–6) acids data because they are nonnormally distributed. Mean ± SD for other data that are normally distributed. Baseline biomarker values were not different between the 2 groups.

3 ANCOVA, adjusted for baseline outcome value and baseline BMI (mean difference in change from baseline, almonds minus control); *P* < 0.05 indicated a significant difference. ANCOVA assumptions were met.

4 *P* < 0.05 indicated a significant difference for values of mean difference between 2 groups. FA, fatty acid.

**TABLE 7:** Fecal SCFAs from a subset of study population following random allocation to almond and control snacks^1^

	Control, *n* = 17		Almonds, *n* = 18		Main comparison between groups^3^
SCFA, μmol/g	Baseline^2^	Change	Baseline^2^	Change	
Acetic acid	50.1 (32.3)	−0.56 (−8.78, 9.90)	49.4 (31.8)	4.61 (−4.47, 13.68)	4.05 (−9.05, 17.15)
Propionic acid	12.8 (7.3)	2.26 (−2.14, 6.66)	13.8 (10.5)	−0.68 (−4.96, 3.59)	−2.94 (−9.13, 3.24)
Isobutyric acid	1.91(0.5)	0.04 (−0.41, 0.50)	1.6 (1.1)	0.27 (−0.18, 0.71)	0.22 (−0.42, 0.87)
Butyric acid	13.6 (8.7)	0.31 (−3.97, 4.59)	13.7 (12.9)	3.06 (−1.10, 7.21)	2.74 (−3.28, 8.77)
Isovaleric acid	2.2 (0.4)	0.10 (−0.43, 0.63)	1.9 (1.4)	0.30 (−0.22, 0.81)	0.20 (−0.55, 0.95)
Valeric acid	1.7 (1.2)	0.11 (−0.22, 0.45)	2.0 (0.8)	0.06 (−0.27, 0.38)	−0.06 (−0.52, 0.41)
Total SCFA	90.1 (52.0)	3.31 (−13.37, 20.0)	85.4 (68.0)	7.66 (−8.55, 23.87)	4.35 (−19.05, 27.75)

^1^Values of change and main comparisons of changes between groups are presented as mean (95% CI) generated from estimated marginal means from ANCOVA.

2 Median (IQR) because data are nonnormally distributed. Baseline biomarker values were not different between the 2 groups.

3 ANCOVA, adjusted for baseline outcome value and baseline BMI (mean difference in change from baseline, almonds minus control). ANCOVA assumptions were met. SCFA, short chain fatty acid.

## Discussion

This study set out to test the hypothesis that substituting whole almonds for typically consumed snacks would increase EDV, decrease liver fat, and improve other markers of cardiometabolic health. Replacing usual snacks with whole almonds, relative to a neutral control snack, caused a 4% unit increase in EDV (FMD) in healthy adults at above-average risk of CVD. According to meta-analyses of clinical studies reporting the predictive value of FMD for cardiovascular events in non-CVD patients, this would be equivalent to an adjusted RR reduction of 32% (pooled RR: 0.92; 95% CI: 0.89, 0.96) per 1% increase in FMD) (27). Thus, simply targeting the quality of snacks eaten between meals can have a measurable beneficial impact on vascular health and would be predicted to significantly reduce risk of CVD. Studies are lacking on the effect of almonds on FMD in healthy individuals (28), although previous meta-analyses of randomized controlled trials of nut consumption (mainly walnuts, but also 2 studies on pistachios, 1 on almonds, and 1 on hazelnuts) in participants with T2D or other health issues have reported smaller effect sizes (0.4–0.8% unit differences) compared with the large difference in the current study (29, 30). The improvement in FMD observed in our current study was not accompanied by an increase in prehyperemia diameter nor endothelium-independent dilation (post-GTN administration), demonstrating that almond consumption had a specific impact on nitric oxide–mediated EDV.

Whole almonds have been shown to reduce postprandial glycemia (31) and lipemia (32) compared with almond products with a high lipid bioaccessibility where lipid has been mechanically released from the plant cell walls. This can attenuate acute increases in oxidative stress and inflammation postprandially (33, 34). Almonds also contain cardioprotective chemicals, including l-arginine [essential in the process of nitric oxide synthesis (33)], phenolic compounds, vitamin E, and folate. Whereas concentrations of intracellular l-arginine already exceed that required for nitric oxide synthesis (33), the increased intake of (poly)phenolics, vitamin E, and folate could reduce oxidative stress and inflammation, thus potentially improving nitric oxide bioavailability (35–39).

Liver fat was a main outcome due to its central role in metabolic regulation and as a predictive factor of CVD and T2D risk (40). No effect of almond consumption was observed on liver fat measured with MRI or ^1^H-MRS (both total percentage and degree of saturation), nor inferred from biochemical markers of liver health (fetuin-A or ALT/GGT). There were relatively low amounts of liver fat in our study population at baseline, with only 10% classified as having fatty liver (liver fat ≥5.5%). Despite participants having higher concentrations of liver fat at baseline (8–10%) in another recent trial, no impact of almond consumption on liver fat was observed (56 g/d almonds compared with isocaloric biscuits over 8 wk; *n* = 36) in participants who were overweight/obese (41). These results demonstrate that almond consumption does not modify liver fat in either direction in a healthy population, despite almonds being a high-fat food. The fact that liver fat did not increase could be due to the fact that on average there was no shift to a positive energy balance and also the low lipid bioaccessibility of almonds (42, 43).

Indicators of insulin sensitivity remained unaffected by the almond intervention. These findings are consistent with previous reports of almonds intakes of 15% of energy or 42–56 g/d in study populations of individuals who were overweight/obese and/or at increased risk of T2D (41, 44–46). The fact that insulin sensitivity was unaffected is perhaps unsurprising considering there were no differences between groups in parameters of ectopic fat, assessed by MRI imaging of visceral, pancreatic, and hepatic fat, and MRS analysis of muscle lipids, nor in anthropometric assessments of body fat. An important aim of the current study was to ensure that both intervention arms were isoenergetic, and the consistency in body weight over time and between treatments demonstrates that this was achieved. Although observational studies report inverse associations between tree nut and almond consumption with BMI and WC (47, 48), average almond intakes are low and therefore these associations are likely to indicate that almond consumers have overall healthier diets leading to lower risk of excess body fat (48). Bowen et al. (41) also reported no significant differences in SAT and VAT measured by MRI/MRS. In contrast, displacement of snacks with 42 g/d almonds for 6 wk in human adults with raised blood concentrations of LDL cholesterol, but who were otherwise healthy (9), lowered abdominal and leg fat measured by DXA despite no differences in body weight. One factor that could influence regional fat distribution is the timing of the snack intake. Evidence for this is provided by results of a 16-wk trial in young Korean adults who consumed 56 g almonds/d immediately before a meal, showing a reduction in visceral fat without changes in body weight, but the same effect was not demonstrated when almonds were consumed >2 h before or after a meal (49). This could relate to a lowering of glycemic and insulinemic responses induced by consumption of almonds immediately before a meal.

Consistent with previous research, we observed a reduction in LDL cholesterol, which is likely to be due to displacement of snacks high in saturated fats with almonds that are rich in unsaturated fats, phytosterols, and fiber. The average reduction in the LDL fraction of 0.25 mmol/L reported here, relative to control, is greater than that reported in a recent meta-analysis of 15 previous almond intervention studies, that is, 0.14 mmol/L (50).

In addition to endothelial function, other measures of cardiovascular function were measured, including BP and HRV. We observed no difference in BP, in contrast to a recent meta-analysis of 15 RCTs (50), which reported a significant reduction in DBP in studies where >42 g almonds (for ≥3 wk) were consumed. A unique finding of the study is the relative increase in the longer-phase HRV parameter VLF during sleep, representing greater parasympathetic regulation at night. Low VLF is predictive of mortality (51) and associated with high biomarkers of systemic inflammation (52). Over 24 h, however, there were no effects on any parameter of HRV. During waking hours, parasympathetically driven longer-phase fluctuations in heart rate (e.g., VLF and SD of the average NN intervals for each 5-min segment of a 24-h HRV recording) in response to neurohormonal and circadian physiological changes are likely to be largely overwhelmed by larger sympathetically driven oscillations generated by physical activities and emotional/psychological influences, unless measured under highly controlled conditions, for example, during supine rest. It was previously reported that 4-wk consumption of pistachios at 20% of EER increased HRV in the resting state in adults with T2D compared with low-fat/high-carbohydrate snacks (53).

A key strength of the current study was the considered design of the control dietary intervention to ensure that treatment effects on cardiometabolic risk factors were not the result of a deterioration in diet quality in the control group. Although the size of the decrease in potassium intake in the control group was larger than the increase in potassium intake in the almond group, all other differences in the changes from baseline for macronutrients and micronutrients were attributable to significant changes in dietary intake in the almond group. Combining measures of liver fat ensured regional variability in hepatic fat distribution was accounted for (by MRI of the whole liver) but small changes could be detected using a more sensitive method (MRS) (54). However, future studies could also benefit from recruiting people with diagnosed fatty liver to enable greater scope for diet-mediated change. Limitations of the study were the fact that there were some differences between groups in cardiometabolic disease risk factors at baseline, despite the minimization of groups for age, sex, ethnicity, and cardiometabolic risk score, and the similarity between groups at baseline for screening variables. The difference in baseline FMD values was unexpected, although the statistical analysis adjusted for differences in baseline values. The imbalance in recruitment by sex could mean that the results might not be as applicable to men because they made up just 30% of the randomized sample. Due to difficulties in obtaining fecal samples, it is likely that SCFA analysis was statistically underpowered. The lack of treatment effect contrasts with results from an in vitro digestion model experiment, where butyrate significantly increased after almond treatment (55). Lastly, there were 25 participants who did not complete usable 4-d food diaries and therefore accurate dietary intake data are missing for nearly one-quarter of the sample population, although inspection of 24-h dietary recalls indicated that compliance did not differ in these participants.

In conclusion, the results of this trial show that replacing typical snacks with almonds can have a meaningful impact on daily nutrient intakes and can improve endothelial function, cardiac autonomic function, and lower LDL cholesterol. However, isoenergetic snack substitution in this trial did not modify regional fat deposition and therefore markers of insulin sensitivity were unaffected. The degree of improvement in endothelial function and LDL cholesterol concentrations suggests that incorporating almonds in the diet in place of typically consumed snacks has the potential to reduce CVD risk by ≤30% and therefore could play a powerful role in enhancing cardiovascular health.

## Supplementary Material

nqaa100_Supplemental_File

## Notes

Funding was received from the Almond Board of California and the Indonesia Endowment Fund for Education. Almonds for the dietary intervention were supplied by the Almond Board of California.

Data described in the manuscript will be made available upon request.

Supplemental Methods, Supplemental Tables 1–5, and Supplemental Figure 1 are available from the “Supplementary data” link in the online posting of the article and from the same link in the online table of contents at https://academic.oup.com/ajcn/.

SEEB and WLH contributed jointly as senior authors.

Abbreviations used: ABP, ambulatory blood pressure; ALT, alanine aminotransferase; ATTIS, Almonds Trial Targeting Dietary Intervention with Snacks; BP, blood pressure; CAIPIRINHA, controlled aliasing in parallel imaging results in higher acceleration; CVD, cardiovascular disease; DBP, diastolic blood pressure; EDV, endothelium-dependent vasodilation; EER, estimated energy requirement; EMCL, extramyocellular lipid; FMD, flow-mediated dilation; FoV, field of view; GGT, γ-glutamyltransferase; GTN, glyceryl trinitrate; HF, high-frequency power; HRV, heart rate variability; IHL, intrahepatic lipid; IMCL, intramyocellular lipid; IPL, intrapancreatic lipid; MR, magnetic resonance; MRS, magnetic resonance spectroscopy; NAFLD, nonalcoholic fatty liver disease; NN, normal-to-normal; RMSSD, root mean square of successive differences of NN intervals; ROI, region of interest; SAT, subcutaneous adipose tissue; SBP, systolic blood pressure; SDNN, standard deviations of the NN intervals; TA, acquisition time; TC, total cholesterol; T2D, type 2 diabetes; VAT, visceral adipose tissue; VLF, very-low-frequency power; WC, waist circumference; 1H-MRS, proton magnetic resonance spectroscopy.
