# Supplementary material for: Snacking on whole almonds for 6 weeks improves endothelial function and lowers LDL cholesterol but does not affect liver fat and other cardiometabolic risk factors in healthy adults: the ATTIS study, a randomized controlled trial
Source: Am J Clin Nutr. 2020 May 15;111(6):1178–89. doi: 10.1093/ajcn/nqaa100 (PMC7266688; doi:10.1093/ajcn/nqaa100)
Supplement: nqaa100_Supplemental_File [file nqaa100_supplemental_file.docx]

**Online Supplementary Material**

**Snacking on whole almonds for 6 weeks improves endothelial function and lowers LDL cholesterol but does not affect liver fat and other cardiometabolic risk factors in healthy adults: the ATTIS study, a randomized controlled trial.**

**Authors:** Dikariyanto V. et al.

**Supplemental Methods**

**Study population**

Recruitment was conducted via advertisements in newspapers (London Metro, London Evening Standard, Camden New Journal, Islington Tribune, Westminster Extra), letters to patients registered at two General Practices (GP) in London, study posters at local libraries, study flyers at community centres and leisure centres throughout London and electronic advertisements (social media, e-mail, GumTree). The recruitment process comprised the dissemination of participant information sheet; the completion of a pre-screening questionnaire, and a screening visit.

Potential participants who were eligible from the pre-screening questionnaire, conducted over the phone or completed on-line, attended a screening visit at King’s College London (KCL) metabolic research unit (MRU) in the fasted state (12h). During the screening visit potential participants completed an eating habit questionnaire and informed consent was taken. Measurements of anthropometry (body height and weight, waist circumference (WC), body mass index (BMI), body fat composition) and clinical blood pressure (cBP) were made. Blood was collected by venepuncture for analysis of blood glucose, full lipids, liver function and full blood count, by Affinity Biomarker Labs (London, UK) using standard clinical chemistry techniques. At the end of the visit, participants were given a 4-d food and activity diary to be completed once they were informed that they were eligible for the study based on the screening results. The inclusion and exclusion criteria are outlined in **Supplemental Table 1.**

**Supplemental Table 1. Inclusion and exclusion criteria**

| Inclusion criteria | Exclusion criteria |
| --- | --- |
| - Men and women - 30-70 y - Regular snack consumer (consume 2 or more snacks per day) - Cardiovascular disease risk score of 2 or more according to Framingham risk score system. To be included individuals had to score a total of 2 or more points in one or more of the following criteria:   (1) total plasma cholesterol (TC) 6.2–7.2 mmol/L (2 points), 5.2–6.1 mmol/L (1 point);  (2) high-density lipoprotein (HDL) cholesterol in men ≤0.9 mmol/L (2 points), 1.0–1.1 (1 point) and women ≤1.1 mmol/L (2 points), 1.2–1.3 mmol/L (1 points);  (3) blood pressure (BP) either systolic BP (SBP) ≥140 mmHg (2 points), 130–139 mmHg (1 point) or diastolic BP (DBP) ≥90 mmHg (2 points);  (4) obesity/adiposity either body mass index (BMI) >30 kg/m^2^ (2 points), 25–30 kg/m^2^ (1 point) for Asian populations [27.5 kg/m^2^ (2 points), 23–27.5 kg/m^2^] (1 point) or waist circumference in men [102 cm (2 points), 94–101 cm (1 point) and women [88 cm (2 points), 80–87 cm (1 point). | - Pre-diabetic or diabetic condition; - Reported history of heart attack (myocardial infarction) or stroke, cancer (excluding basal cell carcinoma) in the last five years, epilepsy or regular fainting, cholestatic liver diseases, pancreatitis, alcohol or drug abuse; - Diagnosis of cardiovascular problems, angina, thrombosis, pacemaker, gastrointestinal disorders, renal or bowel diseases; - Use of a drug likely to alter gastrointestinal motility or nutrient absorption; - Presence of metal inside the body; - Allergy or intolerance to nuts; - Currently pregnant, planning pregnancy, breastfeeding or having given birth in the preceding 12-months; - Unwillingness to follow the protocol and/or give informed consent; - Weight change of >3 kg in preceding 2-months; - BMI of <18 kg/m^2^ or >40 kg/m^2^; - Current smokers or individuals who quit smoking within the last 6-months; - Participation in other research trials involving dietary or drug intervention and/or blood collection in the past 3-months; - Unable or unwilling to comply with study protocol. |

**Formulation of control test meals**

Sweet and savory mini muffin snacks were developed to replicate the average UK snack nutrient profile (**Supplemental** **Table 2)**, which was calculated from snack foods identified in the UK National Diet and Nutrition Survey (NDNS) database (1) (55% energy from available carbohydrate, 36% total fat (14% saturated fat), and 10% protein (2). Prior to commencing the study, a 3-wk feasibility study was conducted and the study verified that the snacks had a neutral effect on lipids, blood pressure and body weight/composition and ensured acceptability of the dietary intervention (2). A blend of plant oils (provided by ADM Oils & Fats Ltd, Erith, UK) was blended with butter to replicate the estimated average fatty acid profile of UK snack-derived fat intake.

**Supplemental Table 2. Recipe and Nutrient composition of control muffins**

|  | Sweet Muffins^1^  (each muffin 21 g and 80 kcal) | Savory muffins^2^  (each muffin 28 g and 80 kcal) |
| --- | --- | --- |
| Blend of plant oils | 3.5 | 3.3 |
| Butter | 0.3 | 0.3 |
| White plain flour | 8.0 | 16.7 |
| White granulated sugar | 7.3 | - |
| Flavouring | 0.3 | - |
| Egg white powder | 1.2 | 0.3 |
| Egg white (liquid) | 5.3 | 3.3 |
| Baking Powder | 0.2 | 0.2 |
| Salt | - | 0.2 |
| Seasoning | - | 0.3 |
| Water | - | 9.3 |
| *Nutrient composition* |  |  |
| Energy, *kcal (kJ) per 100 g* | 380 (1582) | 383 (1600) |
| Fat, *g per 100 g (% Energy)* | 14.8 (35.1%) | 15.8 (37.1%) |
| Carbohydrate, *g per 100 g (% Energy)* | 52 (51.3%) | 50 (49.0%) |
| of which sugars, *g per 100 g (% Energy)* | 29.6 (29.2%) | 0.4 (0.4%) |
| Protein, *g per 100 g (% Energy)* | 8.7 (9.2%) | 8.7 (9.1%) |
| *Fatty acid composition* |  |  |
| MUFA, *g per 100 g (% Energy)* | 6.5 (15.4%) | 6.8 (16.0%) |
| PUFA, *g per 100 g (% Energy)* | 2.2 (5.2%) | 2.5 (5.9%) |
| *Linoleic*, *g per 100 g* | *0.2* | *0.3* |
| *Alpha-linolenic*, *g per 100 g* | *1.9* | *2.2* |
| SFA (g) (palmitic and stearic acid), *g per 100 g (% Energy)* | 5.2 (12.3%) | 5.4 (12.7%) |

^1^Sweet flavours: lemon, orange, banana and caramel

^2^Savory flavours: cheese, garlic and parsley

**Snack provision**

Participants in the control group collected muffins in 2 week intervals and were requested to store them in their freezer immediately. Each morning, participants defrosted their daily portion of control muffins using a microwave or allowed them to get to room temperature throughout the day depending on time of consumption. Almonds were provided as pre-portioned daily packs at 2 week intervals. Participants were provided with 20% estimated energy requirements (EER) from the muffins or almonds which for a 2000 kcal EER, equated to five control muffins daily (400 kcal in (80kcal/muffin)), or 63g/d almonds (100g almonds contains 634 kcal). Comparison of nutritional composition between almonds and control muffins is shown in **Supplemental Table 3**. The ratio of sweet: savory muffins consumed was 70:30, with a mean of 20% of energy from free sugars in total muffins.

**Supplemental Table 3.  Nutritional composition of almonds and control snacks per 400 kcal isocaloric portions.  Values given as 20% of the estimated average requirement for energy (2000 kcal) for adult women.**

| Nutrient | Almonds | Average of sweet and savory control snacks | Control snacks | |
| --- | --- | --- | --- | --- |
|  |  |  | Sweet | Savory |
| Energy, kcal | 400 | 400 | 400 | 400 |
| Protein, %E | 13.4 | 18.0 | 18.0 | 18.0 |
| Carbohydrate, %E | 13.2 | 54.1 | 54.3 | 53.8 |
| Starch, %E | - | 38.7 | 24.5 | 52.9 |
| Sugars, %E | 2.3 | 15.1 | 29.7 | 0.4 |
| Fibre, g | 9.3 | 2.1 | 1.3 | 2.8 |
| Fat, %E | 73.6 | 36.5 | 35.8 | 37.1 |
| SFA, g | 5.6 | 12.8 | 12.6 | 12.8 |
| MUFA, g | 47.9 | 16.0 | 15.8 | 16.0 |
| PUFA, g | 16.0 | 5.6 | 5.4 | 5.9 |
| Sodium, mg | <2.5 | 452.1 | 187.4 | 716.8 |
| Potassium, mg | 463.7 | 162.8 | 145.9 | 179.6 |
| Calcium, mg | 164.7 | 130.1 | 46.1 | 214.1 |
| Magnesium, mg | 181.7 | 19.0 | 15.4 | 22.6 |
| Vitamin E, mg | 14.3 | 1.3 | 1.1 | 1.4 |

**Blood collection and handling procedures**

Blood was collected by trained phlebotomists. At screening, fasting blood was collected by venepuncture using the vacutainer technique for analysis of glucose, lipids, liver function and full blood count. For baseline and endpoint visits, venous cannulation was performed and three samples were collected at 5 minute intervals (0 min, 5 min and 10 min) for analysis of insulin, glucose and non-esterified fatty acid (NEFA) to calculate insulin sensitivity using the Homeostatic Model Assessment of Insulin Resistance (HOMA-IR) method. Plasma lipids (total, HDL-, and LDL-cholesterol and TAG, calculated TC:HDL-C ratio and lipoprotein), fatty liver indicators (fetuin-A and alanine transaminase (ALT)), clinically relevant biomarkers of metabolic dysregulation/insulin resistance (adiponectin, resistin and leptin), and plasma fatty acids, were also obtained from cannulation samples at timepoint 0 min only at baseline and endpoint.

Blood samples were collected using serum, EDTA and sodium fluoride containing tubes. All the blood samples were centrifuged (3000 rpm, 4° C, 15 min; Eppendorf centrifuge 5702 R, Stevenage, UK), except the EDTA tubes from screening (which were kept at room temperature for analysis of full blood count). Screening samples were analysed by the Biochemistry Department, King’s College Hospital on the day of collection. Serum and plasma samples from study days were stored at -80° C and analysed in batches. Fasting blood glucose and non-esterified fatty acids (NEFA) were analysed at King’s College London using an ILAB 650 auto-analyser (Werfen International, Milan, Italy). Insulin, plasma lipids, fetuin-A, ALT, adiponectin, resistin and leptin were analyzed by Affinity Biomarker Labs in London, UK. Metabolomics was measured using high-throughput proton NMR metabolomics (Nightingale Health Ltd, Helsinki, Finland) to quantify routine lipid concentrations within 14 subclasses, fatty acid composition, amino acids, ketone bodies, and other metabolites associated with gluconeogenesis. Details of analysis have been described elsewhere (3).

Plasma fatty acids were analysed by gas liquid chromatography-flame ionization detector (GLC-FID) using a modification of the method detailed by Lepage & Roy (1986) (4). Inside Teflon lined screw capped tubes, 0.1 ml internal standard (1 mg/ml pentadecanoic acid in methanol) and 0.1 ml plasma were added. Into this tube, 2 ml of a prepared methanol/toluene 4:1 volume mixture acidified at 10% volume with acetyl chloride was also added. Following the addition of the fatty acid methyl esters (FAME) reagent, tubes were capped tightly and heated in a water bath for 2 h at 60°C to catalyze transesterification. The mixture was neutralized with 5 ml of 6% w/v aqueous potassium carbonate during the cooling process. Tubes were vortex mixed and centrifuged at 2,500 rpm for 10 min. Supernatant containing the concentrated FAMEs was withdrawn for 0.05 ml into a 250 µl GLC tri-spring glass insert, and this solution was injected into the GLC-FID. FAMEs were separated on an Agilent 6890 Gas Chromatograph fitted with a flame ionisation detector and a 25 m x 220 µm x 0.25 µm BP70 capillary column (SGE). Peaks for the FAMEs were identified by comparison with standard of known composition (fish oil and hemp seed oil).

**Quantification of fecal short chain fatty acids (SCFA) by GLC-FID**

Stool samples were homogenized and stored at -80 °C. SCFA were extracted after the addition of the extraction buffer containing 2,2-dimethyl-butyric acid (internal standard), mercury chloride and phosphoric acid in a ¼ (w/v) dilution (3 g, 12 ml). The fecal slurry was centrifuged at 5000 g for 45 min at 4 °C. The top layer was aspirated by a 5-ml syringe and approximately 1 ml was filtered through a sterile 0.2 μm filter into a GLC vial and stored at -20 °C until analysis.

Calibration was conducted with a 6-component blend of pure SCFA solutions at 4 different concentrations to produce gas-chromatography peak area ratio with respect to internal standard versus SCFA amount plots. Using linear regression analysis, the best equation for the best fit straight line for the calibration curves was obtained.

Extracted SCFA were injected into a 6890 series GLC-FID system (Agilent Technologies, US) equipped with a 220 μm internal diameter, 25 m fused silica capillary column with a film thickness of 0.25 μm (BP21, SGE, AUS). Peaks for each of the SCFA were identified by comparing the equations of the standard curves with the calculated area ratios. Samples were run in duplicate and a 1.2 % formic acid cleaning solution was injected to minimize carry-over from the previous samples. Samples with a variation of more than 5 % were re-analyzed.

**References**

1. Bates B, Lennox A, Prentice A, Bates C, Page P, Nicholson S, Swan G. National Diet and Nutrition Survey: Results from Years 1, 2, 3 and 4 (combined) of the Rolling Programme (2008/2009 – 2011/2012) - Executive summary. London: Public Health England, 2014.
2. Smith L, Dikariyanto V, Francis L, Rokib M, Hall WL, Berry SEE. Estimation of the average nutrient profile of UK snacks and development of a control snack intervention for utilisation in dietary intervention studies. Nutrition Society Summer Conference 2017. London, United Kingdom, 2017 (abstr).
3. Soininen P, Kangas AJ, Wurtz P, Suna T, Ala-Korpela M. Quantitative serum nuclear magnetic resonance metabolomics in cardiovascular epidemiology and genetics. Circ Cardiovasc Genet 2015;8(1):192-206. doi: 10.1161/circgenetics.114.000216.
4. Lepage G, Roy CC. Direct transesterification of all classes of lipids in a one-step reaction. J Lipid Res 1986;27(1):114-120.

**Supplemental Figure 1. CONSORT 2010 Flow Diagram**

Lost to follow-up (n=0)

Discontinued intervention (n=0)

Allocated to control group (n=51)

- Received allocated intervention (n=51)
- Did not receive allocated intervention (n=0)

Lost to follow-up (n=0)

Discontinued intervention (gastrointestinal intolerance of almonds) (n=2)

Allocated to almond group (n=56)

- Received allocated intervention (n=56)
- Did not receive allocated intervention (n=0)

## Allocation

## Follow-Up

Assessed for eligibility (n=294)

Excluded (n=187)

- Not meeting inclusion criteria (n=157)
- Declined to participate (n=10)
- Other reasons (n=20)

Assessed for eligibility after run-in (n=109)

Excluded (n=2)

- Not meeting inclusion criteria (incompliance with the study protocols) (n=1)
- Declined to participate (time commitment issue) (n=1)

Randomized (n=107)

## Enrolment

Analyzed (n_max_=51)

- Included during analysis:
- Nutrient intakes: n=40

(missing data are due to poor quality diet diaries or failure to complete by participants)

- *Vascular function, blood pressure, heart rate variability*

(missing data are due to poor quality read-outs and technological problems)

- Endothelial function via flow-mediated dilation: n=42
- Clinical blood pressure: n=51
- 24-h and day-time blood pressures: n=45
- Night-time blood pressures: n=40
- 24-h SDNN and rMSSD: n=33
- Night-time rMSSD: n=45
- Night-time SDANN: n=45
- Night-time VLF and HF: n=45
- *Body composition and measures of ectopic fat*

(MRI/MRS scanning was planned on a subset of study participants: n = 50 (25 per group. Missing data are due to technical problems)

- BMI: n=45, WC: n=49, Body fat: n=49
- Liver fat and pancreatic fat via MRI: n=26
- Intrahepatic fat; unsaturation, polyunsaturation and saturation indices, via H-MRS: n=22
- Subcutaneous fat via MRI: n=24
- Visceral fat via MRI: n=23
- Intra- and extramyocellular lipid: n=23
- *Circulating biomarkers, plasma fatty acid and metabolomic profiles, fecal short chain fatty acids*

(*Fecal* sample collection was planned on a subset of study participants: n = 30 (15 per group.)

- HOMA-IR, blood glucose, plasma fatty acids and metabolomic profiles: n=48
- Insulin, NEFA, blood lipids, ALT and GGT: n=49
- Leptin, adiponectin, fetuin-A and resistin, n=49
- *Fecal* Short chain fatty acids: n=17

Analyzed (n_max_=54)

- Included during analysis:
- Nutrient intakes: n=40

(missing data are due to poor quality diet diaries or failure to complete by participants)

- *Vascular function, blood pressure, heart rate variability*

(missing data are due to poor quality read-outs and technological issue)

- Endothelial function via flow-mediated dilation: n=47
- Clinical blood pressure: n=54
- 24-h and day-time blood pressures: n=49
- Night-time blood pressures: n=46
- 24-h SDNN and rMSSD: n=29
- Night-time rMSSD: n=50
- Night-time SDANN: n=47
- Night-time VLF and HF: n=47
- *Body composition and measures of ectopic fat*

(MRI/MRS scanning was planned on a subset of study participants: n = 50 (25 per group. Missing data are due to technical problems)

- BMI: n=50, WC: n=51, Body fat: n=52
- Liver fat and pancreatic fat via MRI: n=24
- Intrahepatic fat; unsaturation, polyunsaturation and saturation indices via H-MRS: n=23
- Subcutaneous fat via MRI: n=22
- Visceral fat via MRI: n=22
- Intra- and extramyocellular lipid: n=22
- *Circulating biomarkers, plasma fatty acid and metabolomic profiles, fecal short chain fatty acids*

(*Fecal* sample collection was planned on a subset of study participants: n = 30 (15 per group.)

- HOMA-IR, blood glucose, plasma fatty acids and metabolomic profiles: n=53
- Insulin, NEFA, blood lipids, ALT, GGT: n=53
- Leptin, adiponectin, fetuin-A and resistin: n=52
- *Fecal* short chain fatty acids: n=18

## Analysis

**Supplemental Table 4. Dietary intake**

|  | Control, n_max_=40^1^ | | Almond, n_max_=40^1^ | | *P* value  (time*intervention group) |
| --- | --- | --- | --- | --- | --- |
|  | Baseline | Endpoint | Baseline | Endpoint |  |
| Energy intake, kcal/d | 2088.0 (1927.2, 2248,7) | 1999.8 (1860.9, 2138.6) | 1770.2 (1609.5, 1931.0) | 1767.5 (1628.6, 1906.3) | 0.401 |
| Protein, %E | 15.4 (14.2, 16.5) | 16.0 (-0.5, 1.5) | 15.9 (14.7, 17.1) | 16.8 (15.6, 18.0) | 0.731 |
| Total carbohydrate, %E | 43.3 (41.1, 45.5) | 44.7 (42.5, 46.9) | 41.8 (39.6, 43.9) | 34.5 (32.3, 36.7) | <0.001 |
| Starch, %E | 23.9 (22.3, 25.6) | 26.3 (24.5, 28.1) | 23.5 (21.9, 25.2) | 19.1 (17.3, 20.9) | <0.001 |
| Free sugars, %E | 5.9 (4.9, 7.0) | 6.1 (5.2, 7.0) | 5.5 (4.4, 6.5) | 3.0 (2.1, 3.9) | 0.002 |
| Dietary fibre^1^, g/d | 23.8 (21.6, 25.9) | 21.1 (18.4, 23.8) | 20.8 (18.7, 23.0) | 27.2 (24.5, 29.9) | <0.001 |
| Fat, %E | 36.5 (34.5, 38.5) | 34.1 (32.3, 36.0) | 37.1 (35.1, 39.1) | 45.2 (43.3, 47.0) | <0.001 |
| SFA, %E | 12.3 (11.1, 13.4) | 11.8 (11.0, 12.5) | 12.5 (11.4, 13.7) | 11.1 (10.3, 11.9) | 0.231 |
| MUFA, %E | 11.5 (10.4, 12.6) | 10.8 (9.6, 12.0) | 12.4 (11.3, 13.6) | 20.6 (19.4, 21.8) | <0.001 |
| PUFA, %E | 5.9 (5.2, 6.6) | 5.2 (4.5, 5.8) | 5.9 (5.1, 6.5) | 7.9 (7.2, 8.6) | <0.001 |
| Unsaturated:  saturated fatty acid ratio | 1.5 (1.3, 1.7) | 1.4 (1.2, 1.6) | 1.6 (1.4, 1.8) | 2.7 (2.5, 2.9) | <0.001 |
| Sodium, mg | 2151.8 (1892.6, 2410, 9) | 2245.7 (2042.5, 2448.9) | 1925.5 (1666.4, 2184.7) | 1520.5 (1317.3, 1723.7) | 0.013 |
| Potassium, mg | 3025.4 (2743.0, 3307.9) | 2540.9 (2275.3, 2806.4) | 2538.2 (2255.7, 2820.7) | 2891.6 (2626.0, 3157.2) | <0.001 |
| Calcium, mg | 867.0 (751.9, 982.0) | 832.6 (746.3, 918.8) | 705.2 (590.2, 820.3) | 821.1 (734.8, 907.3) | 0.071 |
| Magnesium, mg | 368.0 (322.9, 413.1) | 297.3 (263.9, 330.7) | 279.2 (234.0, 324.3) | 426.5 (393.1, 459.9) | <0.001 |
| Vitamin E, mg | 10.6 (9.4, 11.9) | 7.9 (6.4, 9.4) | 9.0 (7.7, 10.2) | 23.3 (21.7, 24.8) | <0.001 |
| Riboflavin, mg | 1.8 (1.4, 2.2) | 1.5 (1.3, 1.8) | 1.5 (1.1, 1.9) | 2.0 (1.8, 2.2) | 0.002 |
| Niacin, mg | 16.2 (13.3, 19.0) | 14.1 (12.1, 16.2) | 14.6 (11.7, 17.5) | 14.7 (12.7, 16.7) | 0.227 |

ANCOVA repeated measures was used, adjusted for baseline BMI. ANCOVA assumptions were met.

*P*<0.05 indicating a significant treatment * time interaction.

^1^Data were analyzed using 40 diaries collected from each group. Missing data are due to poor quality diet diaries or failure to complete by participant.

**Supplemental Table 5. Metabolomics data from NMR analysis**

|  | Control, n_max_ = 51^1^ | | Almond, n_max_ = 54^1^ | | Mean comparison between groups^3^ |
| --- | --- | --- | --- | --- | --- |
|  | Baseline^2^ | Change | Baseline^2^ | Change |  |
| Total-C, mmol/L | 4.81 ± 1.08 | -0.01 (-0.25, 0.11) | 4.93 ± 0.89 | -0.15 (-0.33, -0.02) | -0.08 (-0.33, 0.17) |
| VLDL-C, mmol/L | 0.69 ± 0.22 | -0.03 (-0.07, 0.01) | 0.66 ± 0.25 | -0.03 (-0.07, 0.01) | -0.00 (-0.06, 0.05) |
| Remnant-C, mmol/L | 1.43 ± 0.40 | -0.04 (-0.10, 0.03) | 1.41 ± 0.37 | -0.05 (-0.12, 0.01) | -0.02 (-0.11, 0.08) |
| LDL-C, mmol/L | 1.83 ± 0.57 | -0.02 (-0.11, 0.08) | 1.88 ± 0.42 | -0.05 (-0.14, 0.04) | -0.04 (-0.17, 1.00) |
| HDL-C, mmol/L | 1.54 ± 0.39 | -0.02 (-0.08, 0.05) | 1.64 ± 0.44 | -0.04 (-0.10, -0.02) | -0.03 (-0.12, 0.06) |
| HDL2-C, mmol/L | 1.04 ± 0.36 | -0.02 (-0.08, 0.04) | 1.13 ± 0.41 | -0.04 (-0.10, -0.01) | -0.03 (-0.11, 0.06) |
| HDL3-C, mmol/L | 0.50 ± 0.04 | 0.00 (-0.00, 0.01) | 0.51 ± 0.04 | -0.00 (-0.01, 0.00) | -0.01 (-0.01, 0.00) |
| Esterified-C, mmol/L | 3.39 ± 0.78 | -0.05 (-0.19, 0.08) | 3.48 ± 0.64 | -0.10 (-0.23, -0.03) | -0.04 (-0.23, 0.14) |
| Free-C, mmol/L | 1.41 ± 0.30 | -0.02 (-0.07, 0.03) | 1.45 ± 0.25 | -0.04 (-0.08, -0.01) | -0.02 (-0.09, 0.04) |
| Total-TG, mmol/L | 1.14 ± 0.44 | -0.03 (-0.12, 0.06) | 1.09 ± 0.56 | -0.06 (-0.15, 0.02) | -0.03 (-0.15, 0.10) |
| VLDL-TG, mmol/L | 0.71 ± 0.36 | -0.02 (-0.10, 0.05) | 0.68 ± 0.47 | -0.05 (-0.12, 0.03) | -0.02 (-0.13, 0.08) |
| LDL-TG, mmol/L | 0.18 ± 0.05 | -0.00 (-0.01, 0.00) | 0.17 ± 0.47 | -0.01 (-0.01, 0.00) | -0.00 (-0.01, 0.01) |
| HDL-TG, mmol/L | 0.14 ± 0.04 | -0.00 (-0.01, 0.00) | 0.13 ± 0.04 | -0.01 (-0.01, -0.00) | -0.00 (-0.01, 0.01) |
| Phosphoglycerides, mmol/L | 2.16 ± 0.42 | -0.05 (-0.15, 0.04) | 2.21 ± 0.43 | -0.09 (-0.18, 0.00) | -0.03 (-0.16, 0.09) |
| TG:PG | 0.54 ± 0.21 | -0.02 (-0.06, 0.01) | 0.49 ± 0.23 | -0.01 (-0.05, 0.03) | 0.01 (-0.04, 0.07) |
| Cholines, mmol/L | 2.51 ± 0.46 | -0.03 (-0.13, 0.07) | 2.58 ± 0.48 | -0.09 (-0.18, 0.01) | -0.05 (-0.19, 0.09) |
| Phosphatidylcholines, mmol/L | 2.17 ± 0.41 | -0.05 (-0.14, 0.04) | 2.21 ± 0.43 | -0.09 (-0.17, 0.00) | -0.04 (-0.16, 0.09) |
| Sphingomyelins, mmol/L | 0.45 ± 0.10 | -0.00 (-0.02, 0.02) | 0.48 ± 0.08 | -0.02 (-0.04, -0.00) | -0.02 (-0.05, 0.00) |
| Apolipoprotein B, g/L | 0.89 ± 0.20 | -0.01 (-0.04, 0.02) | 0.89 ± 0.18 | -0.02 (-0.06, 0.01) | -0.01 (-0.06, 0.03) |
| Apolipoprotein A1, g/L | 1.60 ± 0.23 | -0.02 (-0.06, 0.02) | 1.65 ± 0.26 | -0.04 (-0.08, 0.00) | -0.02 (-0.08, 0.04) |
| Apolipoprotein B:Apolipoprotein A1 | 0.56 ± 0.13 | -0.00 (-0.02, 0.02) | 0.55 ± 0.14 | -0.00 (-0.02, 0.02) | 0.00 (-0.03, 0.03) |
| Total fatty acids, mmol/L | 12.25 ± 2.15 | -0.32 (-0.80, 0.18) | 12.31 ± 2.54 | -0.53 (-1.00, -0.07) | -0.22 (-0.89, 0.46) |
| Unsaturation degree | 1.23 (0.09) | 0.00 (-0.01, 0.02) | 1.24 (0.10) | -0.01 (-0.01, 0.02) | 0.01 (-0.02, 0.03) |
| Omega-3, mmol/L | 0.52 ± 0.11 | -0.00 (-0.03, 0.03) | 0.52 ± 0.17 | -0.05 (-0.07, -0.02) | -0.04 (-0.08, -0.00)^4^ |
| Omega-6, mmol/L | 4.17 ± 0.74 | -0.10 (-0.25, 0.06) | 4.21 ± 0.72 | -0.14 (-0.29, 0.01) | -0.04 (-0.26, 0.17) |
| Polyunsaturated fatty acids, mmol/L | 4.69 ± 0.83 | -0.10 (-0.28, 0.08) | 4.74 ± 0.85 | -0.19 (-0.36, -0.02) | -0.09 (-0.33, 0.16) |
| Monounsaturated fatty acids, mmol/L | 3.21 ± 0.67 | -0.11 (-0.28, 0.05) | 3.20 ± 0.85 | -0.14 (-0.30, 0.02) | -0.03 (-0.25, 0.20) |
| Saturated fatty acids, mmol/L | 4.34 ± 0.77 | -0.11 (-0.28, 0.07) | 4.38 ± 0.96 | -0.21 (-0.37, -0.04) | -0.10 (-0.39, 0.14) |
| Linoleic acid, mmol/L | 3.62 ± 0.75 | -0.10 (-0.27, 0.07) | 3.64 ± 0.73 | -0.12 (-0.28, 0.04) | -0.02 (-0.25, 0.22) |
| Docosahexaenoic acid, mmol/L | 0.17 ± 0.04 | -0.00 (-0.01, 0.01) | 0.17 ± 0.06 | -0.02 (-0.03, -0.01) | -0.02 (-0.03, -0.00) |
| Alanine, mmol/L | 0.39 ± 0.05 | -0.00 (-0.02, 0.01) | 0.39 ± 0.05 | -0.01 (-0.02, 0.01) | 0.00 (-0.02, 0.02) |
| Glutamine, mmol/L | 0.52 ± 0.06 | -0.02 (-0.03, -0.01) | 0.051 ± 0.05 | -0.01 (-0.02, 0.01) | 0.01 (-0.01, 0.03) |
| Glycine, mmol/L | 0.26 ± 0.05 | -0.00 (-0.01, 0.00) | 0.27 ± 0.06 | -0.01 (-0.02, 0.00) | -0.00 (-0.02, 0.01) |
| Histidine, mmol/L | 0.07 ± 0.01 | 0.20 (0.18, 0.22) | 0.07 ± 0.01 | 0.19 (0.17, 0.21) | -0.01 (-0.04, 0.02) |
| Isoleucine, mmol/L | 0.05 ± 0.01 | 0.00 (-0.00, 0.00) | 0.05 ± 0.01 | -0.00 (-0.01, 0.00) | -0.00 (-0.01, 0.00) |
| Leucine, mmol/L | 0.07 ± 0.01 | 0.00 (-0.00, 0.00) | 0.07 ± 0.01 | -0.00 (-0.00, 0.00) | -0.00 (-0.01, 0.00) |
| Valine, mmol/L | 0.16 ± 0.03 | 0.01 (-0.00, 0.01) | 0.15 ± 0.04 | -0.00 (-0.01, 0.01) | -0.01 (-0.02, 0.00) |
| Phenylalanine, mmol/L | 0.07 ± 0.01 | 0.00 (-0.00, 0.00) | 0.07 ± 0.01 | -0.00 (-0.00, 0.00) | -0.00 (-0.00, 0.00) |
| Tyrosine, mmol/L | 0.05 ± 0.01 | 0.00 (-0.00, 0.00) | 0.05 ± 0.01 | -0.00 (-0.00, 0.00) | -0.00 (-0.00, 0.00) |
| Glucose, mmol/L | 3.96 ± 0.39 | -0.04 (-0.14, 0.05) | 3.89 ± 0.40 | -0.00 (-0.09, 0.09) | 0.04 (-0.09, 0.17) |
| Lactate, mmol/L | 1.28 ± 0.33 | 0.04 (-0.06, 0.14) | 1.37 ± 0.39 | -0.02 (-0.11, 0.08) | -0.06 (-0.20, 0.09) |
| Pyruvate, mmol/L | 0.07 ± 0.02 | 0.00 (-0.00, 0.01) | 0.07 ± 0.02 | 0.00 (-0.01, 0.01) | -0.00 (-0.01, 0.01) |
| Citrate, mmol/L | 0.12 ± 0.02 | -0.01 (-0.01, 0.00) | 0.12 ± 0.02 | 0.00 (-0.00, 0.01) | 0.01 (-0.00, 0.02)^4^ |
| Glycerol, mmol/L | 0.08 ± 0.03 | -0.01 (-0.02, -0.00) | 0.09 ± 0.03 | -0.00 (-0.01, 0.00) | 0.01 (-0.00, 0.02) |
| Acetate, mmol/L | 0.04 ± 0.01 | -0.00 (-0.01, 0.00) | 0.05 ± 0.02 | 0.00 (-0.00, 0.01) | 0.00 (-0.00, 0.01) |
| Acetoacetate, mmol/L | 0.04 (0.02) | 0.00 (-0.01, 0.01) | 0.03 (0.02) | 0.00 (-0.00, 0.01) | 0.00 (-0.01, 0.01) |
| Beta-hydroxybutyrate, mmol/L | 0.16 ± 0.08 | -0.01 (-0.03, 0.02) | 0.16 ± 0.10 | 0.01 (-0.01, 0.03) | 0.02 (-0.01, 0.05) |
| Creatinine, mmol/L | 0.06 ± 0.01 | 0.00 (-0.00, 0.00) | 0.06 ± 0.01 | 0.00 (-0.00, 0.00) | 0.00 (-0.00, 0.00) |
| Albumin, signal area | 0.10 ± 0.01 | -0.00 (-0.01, 0.00) | 0.10 ± 0.01 | -0.00 (-0.01, -0.00) | -0.00 (-0.00, 0.00) |
| Glycoprotein acetyls, mmol/L | 1.33 ± 0.17 | -0.05 (-0.09, 0.01) | 1.31 ± 0.20 | -0.03 (-0.07, 0.00) | 0.01 (-0.04, 0.07) |
| XXL-VLDL-P, µmol/L | 0.00 (0.00) | -0.00 (-0.00, 0.00) | 0.00 (0.00) | -0.00 (-0.00, 0.00) | -0.00 (-0.00, 0.00) |
| XXL-VLDL-L, µmol/L | 12.60 (18.15) | -0.42 (-4.00, 3.17) | 8.04 (20.09) | -1.70 (-5.12, 1.71) | -1.29 (-6.24, 3.67) |
| XXL-VLDL-PL, µmol/L | 1.41 (2.32) | -0.06 (-0.51, 0.39) | 0.87 (2.37) | -0.22 (-0.66, 0.21) | -0.16 (-0.79, 0.46) |
| XXL-VLDL-C, µmol/L | 2.27 (3.77) | 0.04 (-0.58, 0.66) | 1.15 (3.53) | -0.35 (-0.94, 0.24) | -0.39 (-1.24, 0.47) |
| XXL-VLDL-CE, µmol/L | 1.58 (2.90) | 0.04 (-0.33, 0.41) | 1.08 (2.24) | -0.20 (-0.56, 0.15) | -0.24 (-0.76, 0.27) |
| XXL-VLDL-FC, µmol/L | 0.67 (1.22) | -0.00 (-0.27, 0.26) | 0.19 (1.32) | -0.15 (-0.39, 0.10) | -0.14 (-0.50, 0.22) |
| XXL-VLDL-TG, µmol/L | 9.03 (12.14) | -0.40 (-2.94, 2.15) | 6.50 (13.85) | -1.13 (-3.55, 1.30) | -0.73 (-4.24, 2.78) |
| XL-VLDL-P, µmol/L | 0.00 (0.00) | 0.00 (-0.00, 0.00) | 0.00 (0.00) | 0.00 (0.00, 0.00) | 0.00 (0.00, 0.00) |
| XL-VLDL-L, µmol/L | 31.48 (49.16) | -1.50 (-10.43, 7.43) | 13.25 (53.94) | -5.39 (-13.89, 3.11) | -3.89 (-16.22, 8.44) |
| XL-VLDL-PL, µmol/L | 4.75 (8.25) | -0.18 (-1.67, 1.31) | 2.58 (9.78) | -0.91 (-2.33, 0.51) | -0.73 (-2.79, 1.33) |
| XL-VLDL-C, µmol/L | 6.06 (7.67) | -0.04 (-1.74, 1.67) | 3.18 (10.11) | -0.89 (-2.52, 0.73) | -0.86 (-3.22, 1.50) |
| XL-VLDL-CE, µmol/L | 3.43 (4.55) | -0.09 (-1.02, 0.84) | 1.89 (5.54) | -0.51 (-1.40, 0.37) | -0.43 (-1.71, 0.86) |
| XL-VLDL-FC, µmol/L | 2.21 (3.83) | 0.05 (-0.74, 0.84) | 1.21 (4.42) | -0.38 (-1.13, 0.37) | -0.43 (-1.52, 0.66) |
| XL-VLDL-TG, µmol/L | 19.37 (31.96) | -1.29 (-7.10, 4.53) | 9.60 (33.90) | -3.59 (-9.12, 1.94) | -2.30 (-10.33, 5.72) |
| L-VLDL-P, µmol/L | 0.00 ± 0.00 | 0.00 (-0.00, 0.00) | 0.00 ± 0.00 | -0.00 (-0.00, 0.00) | 0.00 (-0.00, 0.00) |
| L-VLDL-L, µmol/L | 194.63 ± 147.08 | -8.02 (-39.91, 23.87) | 182.98 ± 199.46 | -20.23 (-50.58, 10.12) | -12.21 (-56.24, 31.81) |
| L-VLDL-PL, µmol/L | 36.56 ± 26.85 | -1.45 (-7.25, 4.36) | 34.40 ± 36.70 | -3.81 (-9.33, 1.71) | -2.36 (-10.37, 5.64) |
| L-VLDL-C, µmol/L | 46.38 ± 34.71 | -2.55 (-9.87, 4.77) | 42.63 ± 46.13 | -5.11 (-12.07, 1.86) | -2.56 (-12.67, 7.55) |
| L-VLDL-CE, µmol/L | 28.08 ± 18.94 | -1.92 (-5.84, 2.01) | 25.23 ± 24.41 | -2.74 (-6.48, 0.99) | -0.82 (-6.25, 4.60) |
| L-VLDL-FC, µmol/L | 18.30 ± 16.06 | -0.64 (-4.15, 2.86) | 17.40 ± 22.06 | -2.35 (-5.69, 0.98) | -1.71 (-6.55, 3.13) |
| L-VLDL-TG, µmol/L | 111.68 ± 86.02 | -4.03 (-22.87, 14.81) | 105.95 ± 116.83 | -11.31 (-29.24, 6.61) | -7.29 (-33.29, 18.72) |
| M-VLDL-P, µmol/L | 0.01 ± 0.01 | -0.00 (-0.00, 0.00) | 0.01 ± 0.01 | -0.00 (-0.00, 0.00) | 0.00 (-0.00, 0.00) |
| M-VLDL-L, µmol/L | 498.84 ± 241.84 | -20.92 (-70.71, 28.87) | 466.69 ± 313.19 | -30.77 (-78.15, 16.61) | -9.85 (-78.62, 58.92) |
| M-VLDL-PL, µmol/L | 103.89 ± 47.55 | -4.50 (-14.20, 5.20) | 97.06 ± 61.42 | -6.06 (-15.29, 3.16) | -1.56 (-14.96, 11.83) |
| M-VLDL-C, µmol/L | 150.28 ± 66.03 | -8.66 (-21.92, 4.60) | 139.01 ± 84.02 | -10.04 (-22.66, 2.58) | -1.38 (-19.70, 16.94) |
| M-VLDL-CE, µmol/L | 95.03 ± 37.24 | -6.31 (-13.59, 0.98) | 88.08 ± 45.92 | -5.76 (-12.69, 1.18) | 0.55 (-9.53, 10.62) |
| M-VLDL-FC, µmol/L | 55.25 ± 30.99 | -2.36 (-8.66, 3.94) | 50.92 ± 39.27 | -4.27 (-10.26, 1.73) | -1.91 (-10.61, 6.80) |
| M-VLDL-TG, µmol/L | 244.67 ± 132.86 | -7.83 (-35.21, 19.54) | 230.62 ± 169.70 | -14.60 (-40.65, 11.44) | -6.77 (-44.57, 31.03) |
| S-VLDL-P, µmol/L | 0.03 ± 0.01 | -0.00 (-0.00, 0.00) | 0.03 ± 0.01 | -0.00 (-0.00, 0.00) | -0.00 (-0.00, 0.00) |
| S-VLDL-L, µmol/L | 580.88 ± 191.1 | -27.60 (-64.01, 8.81) | 548.19 ± 222.80 | -28.79 (-63.43, 5.86) | -1.19 (-51.51, 49.14) |
| S-VLDL-PL, µmol/L | 138.07 ± 41.15 | -6.75 (-14.87, 1.38) | 131.3 ± 48.10 | -6.55 (-14.28, 1.18) | 0.20 (-11.03, 11.43) |
| S-VLDL-C, µmol/L | 230.10 ± 74.32 | -12.11 (-25.21, 0.99) | 219.37 ± 74.03 | -11.98 (-24.44, 0.49) | 0.14 (-17.97, 18.24) |
| S-VLDL-CE, µmol/L | 148.56 ± 50.20 | -7.67 (-16.27, 0.94) | 142.47 ± 45.04 | -7.60 (-15.78, 0.59) | 0.07 (-11.82, 11.95) |
| S-VLDL-FC, µmol/L | 81.54 ± 27.15 | -4.35 (-9.57, 0.87) | 76.90 ± 30.98 | -4.46 (-9.43, 0.51) | -0.11 (-7.32, 7.11) |
| S-VLDL-TG, µmol/L | 212.71 ± 88.79 | -8.60 (-26.09, 8.89) | 197.51 ± 106.88 | -10.39 (-27.03, 6.26) | -1.79 (-25.96, 22.39) |
| XS-VLDL-P, µmol/L | 0.04 ± 0.01 | -0.00 (-0.00, 0.00) | 0.04 ± 0.01 | -0.00 (-0.00, 0.00) | 0.00 (-0.00, 0.00) |
| XS-VLDL-L, µmol/L | 580.88 ± 191.10 | -27.60 (-64.01, 8.81) | 548.19 ± 222.80 | -28.79 (-63.43, 5.86) | -1.19 (-51.51, 49.14) |
| XS-VLDL-PL, µmol/L | 168.71 ± 44.70 | -3.49 (-10.03, 3.04) | 169.42 ± 36.26 | -4.61 (-10.83, 1.60) | -1.12 (-10.14, 7.90) |
| XS-VLDL-C, µmol/L | 252.46 ± 70.37 | -6.10 (-17.02, 4.83) | 249.80 ± 55.10 | -6.64 (-17.04, 3.76) | -0.54 (-15.63, 14.54) |
| XS-VLDL-CE, µmol/L | 164.11 ± 48.72 | -3.81 (-12.16, 4.55) | 161.92 ± 38.38 | -4.16 (-12.11, 3.79) | -0.35 (-11.89, 11.18) |
| XS-VLDL-FC, µmol/L | 88.35 ± 22.58 | -2.26 (-5.63, 1.11) | 87.88 ± 18.90 | -2.51 (-5.72, 0.70) | -0.25 (-4.90, 4.41) |
| XS-VLDL-TG, µmol/L | 106.78 ± 32.40 | -3.75 (-9.98, 2.48) | 101.59 ± 38.10 | -3.91 (-9.84, 2.02) | -0.16 (-8.76, 8.45) |
| IDL-P, µmol/L | 0.11 ± 0.03 | -0.00 (-0.01, 0.00) | 0.12 ± 0.02 | -0.00 (-0.01, 0.00) | -0.00 (-0.01, 0.01) |
| IDL-L, µmol/L | 1167.57 ± 321.88 | -12.08 (-61.54, 37.37) | 1181.56 ± 237.80 | -32.55 (-79.61, 14.51) | -20.46 (-88.74, 47.81) |
| IDL-PL, µmol/L | 317.71 ± 82.31 | -320.65 (-320.65, -320.65) | 322.24 ± 59.41 | -320.65 (-320.65, -320.65) | -0.00 (-0.00, 0.00) |
| IDL-C, µmol/L | 737.57 ± 219.79 | -7.01 (-42.56, 28.54) | 749.88 ± 158.88 | -21.09 (-54.93, 12.74) | -14.09 (-63.17, 35.00) |
| IDL-CE, µmol/L | 522.63 ± 156.19 | -5.17 (-31.28, 20.95) | 529.62 ± 113.91 | -14.94 (-39.79, 9.91) | -9.77 (-45.82, 26.28) |
| IDL-FC, µmol/L | 214.94 ± 64.42 | -1.88 (-11.54, 7.78) | 220.26 ± 46.52 | -6.12 (-15.31, 3.07) | -4.24 (-17.58, 9.09) |
| IDL-TG, µmol/L | 112.29 ± 29.54 | -2.20 (-7.20, 2.80) | 109.44 ± 31.53 | -3.46 (-8.22, 1.30) | -1.26 (-8.17, 5.65) |
| L-LDL-P, µmol/L | 0.19 ± 0.05 | -0.00 (-0.01, 0.01) | 0.20 ± 0.04 | -0.01 (-0.01, 0.00) | -0.00 (-0.02, 0.01) |
| L-LDL-L, µmol/L | 1389.83 ± 390.38 | -15.25 (-78.15, 47.66) | 1413.76 ± 290.57 | -41.03 (-100.89, 18.83) | -25.79 (-112.63, 61.06) |
| L-LDL-PL, µmol/L | 346.55 ± 81.45 | -3.61 (-17.07, 9.84) | 352.32 ± 60.57 | -8.67 (-21.48, 4.14) | -5.06 (-23.64, 13.52) |
| L-LDL-C, µmol/L | 945.01 ± 288.55 | -9.99 (-57.42, 37.45) | 964.84 ± 211.22 | -29.03 (-74.17, 16.11) | -19.04 (-84.53, 46.44) |
| L-LDL-CE, µmol/L | 679.47 ± 217.08 | -8.22 (-44.28, 27.84) | 693.23 ± 160.83 | -22.48 (-56.79, 11.83) | -14.26 (-64.04, 35.52) |
| L-LDL-FC, µmol/L | 265.53 ± 72.05 | -1.78 (-13.29, 9.73) | 271.61 ± 51.50 | -6.54 (-17.50, 4.41) | -4.76 (-20.66, 11.14) |
| L-LDL-TG, µmol/L | 98.28 ± 26.13 | -1.70 (-5.73, 2.34) | 96.61 ± 25.36 | -3.29 (-7.12, 0.55) | -1.59 (-7.16, 3.98) |
| M-LDL-P, µmol/L | 0.16 ± 0.05 | -0.00 (-0.01, 0.01) | 0.16 ± 0.03 | -0.00 (-0.01, 0.00) | -0.00 (-0.01, 0.01) |
| M-LDL-L, µmol/L | 813.94 ± 231.79 | -9.44 (-47.50, 28.62) | 829.79 ± 174.64 | -22.79 (-59.01, 13.43) | -13.35 (-65.89, 39.20) |
| M-LDL-PL, µmol/L | 210.96 ± 44.65 | -2.68 (-10.31, 4.95) | 214.04 ± 35.36 | -5.06 (-12.32, 2.20) | -2.38 (-12.91, 8.15) |
| M-LDL-C, µmol/L | 553.86 ± 177.62 | -6.02 (-35.58, 23.54) | 567.16 ± 130.66 | -15.98 (-44.11, 12.15) | -9.96 (-50.77, 30.85) |
| M-LDL-CE, µmol/L | 402.73 ± 145.10 | -4.68 (-28.74, 19.39) | 412.95 ± 106.87 | -13.01 (-35.92, 9.89) | -8.34 (-41.56, 24.89) |
| M-LDL-FC, µmol/L | 151.13 ± 32.81 | -1.35 (-6.93, 4.23) | 154.21 ± 24.31 | -2.96 (-8.27, 2.35) | -1.61 (-9.32, 6.10) |
| M-LDL-TG, µmol/L | 49.12 ± 13.00 | -0.78 (-2.87, 1.32) | 48.59 ± 12.76 | -1.72 (-3.72, 0.27) | -0.95 (-3.84, 1.95) |
| S-LDL-P, µmol/L | 0.18 ± 0.05 | -0.00 (-0.01, 0.01) | 0.19 ± 0.04 | -0.01 (-0.01, 0.00) | -0.00 (-0.01, 0.01) |
| S-LDL-L, µmol/L | 518.43 ± 139.83 | -6.61 (-29.51, 16.29) | 531.48 ± 107.35 | -14.51 (-36.30, 7.29) | -7.90 (-39.52, 23.73) |
| S-LDL-PL, µmol/L | 152.63 ± 28.38 | -2.19 (-7.27, 2.89) | 155.84 ± 23.68 | -3.71 (-8.55, 1.12) | -1.52 (-8.54, 5.49) |
| S-LDL-C, µmol/L | 335.88 ± 106.68 | -3.65 (-21.03, 13.73) | 346.06 ± 78.45 | -9.26 (-25.80, 7.28) | -5.61 (-29.61, 18.39) |
| S-LDL-CE, µmol/L | 245.39 ± 87.55 | -2.70 (-16.76, 11.36) | 253.25 ± 64.01 | -7.42 (-20.81, 5.96) | -4.72 (-24.14, 14.69) |
| S-LDL-FC, µmol/L | 90.49 ± 19.54 | -0.96 (-4.38, 2.45) | 92.82 ± 14.93 | -1.83 (-5.08, 1.42) | -0.87 (-5.59, 3.85) |
| S-LDL-TG, µmol/L | 29.92 ± 8.35 | -0.82 (-2.38, 0.75) | 29.57 ± 9.60 | -1.49 (-2.98, 0.00) | -0.67 (-2.83, 1.49) |
| XL-HDL-P, µmol/L | 0.41 ± 0.25 | -0.00 (-0.05, 0.04) | 0.49 ± 0.27 | -0.03 (-0.08, 0.01) | -0.03 (-0.09, 0.04) |
| XL-HDL-L, µmol/L | 413.02 ± 257.10 | -2.67 (-49.96, 44.62) | 494.59 ± 276.00 | -31.38 (-76.36, 13.60) | -28.71 (-94.39, 36.97) |
| XL-HDL-PL, µmol/L | 225.98 ± 140.62 | -6.19 (-31.18, 18.80) | 266.78 ± 151.56 | -18.81 (-42.58, 4.95) | -12.62 (-47.29, 22.05) |
| XL-HDL-C, µmol/L | 174.66 ± 115.95 | 2.98 (-20.27, 26.24) | 215.50 ± 124.71 | -10.33 (-32.44, 11.78) | -13.31 (-45.64, 19.02) |
| XL-HDL-CE, µmol/L | 125.45 ± 81.85 | 2.37 (-14.83, 19.58) | 155.38 ± 87.79 | -6.61 (-22.97, 9.75) | -8.98 (-32.91, 14.95) |
| XL-HDL-FC, µmol/L | 49.21 ± 34.66 | 0.42 (-0.01, 0.01) | 60.12 ± 37.62 | -0.00 (-0.01, 0.00) | -0.00 (-0.01, 0.01) |
| XL-HDL-TG, µmol/L | 12.39 ± 7.63 | 0.00 (-5.90, 6.75) | 12.31 ± 7.69 | -3.55 (-9.57, 2.47) | -3.97 (-12.75, 4.81) |
| L-HDL-P, µmol/L | 1.26 ± 0.58 | -0.03 (-0.12, 0.06) | 1.37 ± 0.66 | -0.07 (-0.16, 0.01) | -0.04 (-0.16, 0.08) |
| L-HDL-L, µmol/L | 790.73 ± 374.02 | -19.63 (-76.59, 37.33) | 864.08 ± 426.66 | -45.53 (-99.73, 8.66) | -25.90 (-104.69, 52.89) |
| L-HDL-PL, µmol/L | 393.74 ± 165.70 | -12.86 (-39.86, 14.13) | 425.37 ± 188.47 | -21.12 (-46.80, 4.57) | -8.25 (-45.58, 29.08) |
| L-HDL-C, µmol/L | 367.09 ± 199.49 | -5.81 (-34.97, 23.35) | 410.20 ± 228.83 | -21.90 (-49.64, 5.85) | -16.09 (-56.44, 24.27) |
| L-HDL-CE, µmol/L | 286.67 ± 152.02 | -4.73 (-27.22, 17.75) | 318.90 ± 174.22 | -16.92 (-38.31, 4.47) | -12.19 (-43.30, 18.92) |
| L-HDL-FC, µmol/L | 80.42 ± 47.61 | -1.09 (-7.82, 5.65) | 91.30 ± 54.70 | -4.96 (-11.37, 1.44) | -3.88 (-13.20, 5.44) |
| L-HDL-TG, µmol/L | 29.89 ± 15.46 | -0.94 (-3.17, 1.30) | 28.51 ± 13.56 | -2.54 (-4.67, -0.42) | -1.61 (-4.69, 1.47) |
| M-HDL-P, µmol/L | 2.23 ± 0.42 | -0.07 (-0.16, 0.02) | 2.27 ± 0.43 | -0.06 (-0.15, 0.02) | 0.00 (-0.11, 0.13) |
| M-HDL-L, µmol/L | 947.62 ± 182.64 | -29.32 (-66.70, 8.07) | 965.17 ± 190.69 | -26.62 (-62.19, 8.96) | 2.70 (-48.92, 54.32) |
| M-HDL-PL, µmol/L | 427.40 ± 77.79 | -11.68 (-27.93, 4.57) | 433.73 ± 79.36 | -12.26 (-27.73, 3.20) | -0.58 (-23.02, 21.86) |
| M-HDL-C, µmol/L | 473.24 ± 104.61 | -15.27 (-35.85, 5.32) | 487.75 ± 113.80 | -12.18 (-31.77, 7.41) | 3.09 (-25.35, 31.52) |
| M-HDL-CE, µmol/L | 385.83 ± 83.54 | -11.97 (-28.22, 4.29) | 397.98 ± 91.43 | -9.30 (-24.77, 6.17) | 2.67 (-19.79, 25.12) |
| M-HDL-FC µmol/L | 87.41 ± 21.48 | -3.29 (-7.68, 1.11) | 89.77 ± 22.72 | -2.89 (-7.07, 1.29) | 0.40 (-5.67, 6.47) |
| M-HDL-TG, µmol/L | 46.98 ± 13.61 | -2.28 (-4.91, 0.35) | 43.69 ± 14.12 | -2.25 (-4.91, 0.35) | 0.03 (-3.61, 3.67) |
| S-HDL-P, µmol/L | 5.22 ± 0.54 | -0.09 (-0.21, 0.03) | 5.17 ± 0.52 | -0.06 (-0.17, 0.05) | 0.03 (-0.14, 0.19) |
| S-HDL-L, µmol/L | 1161.61 ± 119.48 | -19.61 (-46.43, 7.21) | 1152.75 ± 115.44 | -13.27 (-38.78, 12.25) | 6.34 (-30.71, 43.40) |
| S-HDL-PL, µmol/L | 589.38 ± 79.88 | -10.79 (-24.87, 3.29) | 582.77 ± 68.98 | -8.59 (-21.99, 4.81) | 2.20 (-17.25, 21.65) |
| S-HDL-C, µmol/L | 524.87 ± 68.68 | -8.23 (-25.36, 8.91) | 526.60 ± 70.77 | -3.45 (-19.76, 12.86) | 4.78 (-18.88, 28.44) |
| S-HDL-CE, µmol/L | 411.51 ± 64.73 | -5.93 (-21.57, 9.70) | 414.29 ± 64.61 | -1.65 (-16.52, 13.23) | 4.29 (-17.30, 25.87) |
| S-HDL-FC, µmol/L | 113.37 ± 14.55 | -2.36 (-5.11, 0.39) | 112.31 ± 13.08 | -1.74 (-4.36, 0.89) | 0.62 (-3.18, 4.42) |
| S-HDL-TG, µmol/L | 47.36 ± 14.47 | -1.11 (-3.47, 1.26) | 43.38 ± 16.12 | -0.77 (-3.01, 1.48) | 0.34 (-2.93, 3.61) |
| VLDL size, nm | 36.10 ± 1.25 | -0.16 (-0.39, 0.06) | 35.84 ± 1.32 | -0.08 (-0.30, 0.14) | 0.08 (-0.23, 0.40) |
| LDL size, nm | 23.51 ± 0.10 | 0.01 (-0.01, 0.02) | 23.50 ± 0.06 | -0.00 (-0.02, 0.01) | -0.01 (-0.03, 0.02) |
| HDL size, nm | 9.94 ± 0.29 | 0.01 (-0.03, 0.05) | 10.00 ± 0.30 | -0.03 (-0.07, 0.01) | -0.04 (-0.10, 0.01) |

Values of change and main comparison between groups are presented as mean (95% CI)

^1^Not all data were analysed due to technical problems and sample loss.

VLDL-C, esterified-C, free-C, phosphoglycerides, TG:PG, cholines, phosphatidylcholines, sphingomyelins, total fatty acids, unsaturation, omega-3, omega-6, polyunsaturated fatty acids, monounsaturated fatty acids, saturated fatty acids, linoleic acid, docosahexaenoic acid: n = 48 (control) and 52 (almond)

Glutamine: n = 48 (control) and 50 (almond)

Histidine: n = 25 (control) and 33 (almond)

Other biomarkers: n = 48 (control) and 53 (almond)

^2^Mean ± SD for normal distributed data or median (IQR) for non-normally distributed data. Baseline biomarker values were not different between the two groups.

^3^ANCOVA, adjusted for baseline outcome value and baseline BMI (mean difference almonds – control); *P* < 0.05 indicating a significant difference.

^4^*P* < 0.05 indicating a significant difference for values of mean difference between two groups.

C, cholesterol; Remnant-C, TG, triglycerides; PG, phosphoglycerides; VLDL, very low-density lipoprotein; LDL, low-density lipoprotein; HDL, high-density lipoprotein; IDL, intermediate density-lipoprotein; remnant cholesterol (non-HDL, non-LDL -cholesterol); sizes of lipoprotein: XXL – chylomicrons and extremely large, XL – very large, L – large, M – medium, S – small, XS – very small; P, particles; L, total lipids; PL, phospholipids; CE, cholesteryl esters; FC, free cholesterol; XXL-VLDL-P, concentration of chylomicrons and extremely large VLDL particles; XXL-VLDL-L, total lipids in chylomicrons and extremely large VLDL; XXL-VLDL-PL, phospholipids in chylomicrons and extremely large VLDL; XXL-VLDL-C, cholesterol in chylomicrons and extremely large VLDL; XXL-VLDL-CE, cholesteryl esters in chylomicrons and extremely large VLDL; XXL-VLDL-FC, free cholesterol in chylomicrons and extremely large VLDL; XXL-VLDL-TG; triglycerides in chylomicrons and extremely large VLDL; and so on according to size and density of lipoproteins.
